# Naïve, unenculturated chimpanzees fail to make and use flaked stone tools

**DOI:** 10.12688/openreseurope.13186.1

**Published:** 2021-03-24

**Authors:** Elisa Bandini, Alba Motes-Rodrigo, William Archer, Tanya Minchin, Helene Axelsen, Raquel Adriana Hernandez-Aguilar, Shannon P. McPherron, Claudio Tennie

**Affiliations:** 1 Department for Early Prehistory and Quaternary Ecology, University of Tübingen, Tübingen, 72070, Germany; 2 Department of Human Evolution, Max Planck Institute for Evolutionary Anthropology, Leipzig, 04103, Germany; 3 Kristiansand Zoo, Kardemomme By, Kristiansand, 4609, Norway; 4 Department of Social Psychology and Quantitative Psychology, University of Barcelona, Barcelona, 08035, Spain; 5 Centre for Ecological and Evolutionary Synthesis, University of Oslo, Oslo, NO-0316, Norway

**Keywords:** chimpanzee flaking, chimpanzee tool use, lithic technologies, hominid material culture

## Abstract

**Background**: Despite substantial research on early hominin lithic technologies, the learning mechanisms underlying flake manufacture and use are contested. To draw phylogenetic inferences on the potential cognitive processes underlying the acquisition of both of these abilities in early hominins, we investigated if and how one of our closest living relatives, chimpanzees ( *Pan troglodytes*), could learn to make and use flakes.

**Methods**: Across several experimental conditions, we tested unenculturated, naïve chimpanzees from two independent populations (n=11) for their abilities to spontaneously make and use their own flakes as well as to use pre-made flakes made by a human experimenter.

**Results**: Despite the fact that the chimpanzees demonstrated an understanding of the requirements of the task and that subjects were sufficiently motivated and had ample opportunities to develop these behaviours, none of the chimpanzees tested, made or used flakes in any of the experimental conditions.

**Conclusions**: These results differ from all previous ape flaking experiments, which found flake manufacture and use in bonobos and one orangutan. However, these earlier studies tested human-enculturated apes and provided the test subjects with flake making and using demonstrations. The contrast between these earlier positive findings and our negative findings (despite using a much larger sample size) suggests that human enculturation and/or human demonstrations may be necessary for chimpanzees to acquire these abilities. The data obtained in this study are consistent with the hypothesis that flake manufacture and use might have evolved in the hominin lineage after the split between *Homo* and *Pan* 7 million years ago, a scenario further supported by the initial lack of flaked stone tools in the archaeological record after this split. We discuss possible evolutionary scenarios for flake manufacture and use in both non-hominin and hominin lineages.

## Plain language summary

One of the first types of tools found in the archaeological record are sharp-edged stones. How tool-using hominins learnt to make and use these tools is still debated. One way to study how hominins might have learnt to make and use stone tools is to test our closest living relatives, chimpanzees. In this study we provided 11 untrained chimpanzees housed at two different zoological institutions with the motivation to make sharp stone tools (a puzzle box with food rewards that could only be opened with a sharp tool) and stone cores and hammerstones that the chimpanzees could use to make the stone tools necessary to open the puzzle box. No demonstrations on how to make or use stone tools were provided to the chimpanzees before or during testing. Although the chimpanzees were motivated and interested in accessing the puzzle box, none of the subjects in this study made or used sharp stones. Therefore, these findings suggest that without extensive human training and demonstrations, chimpanzees cannot learn how to make or use stone tools by themselves. This data is consistent with the hypothesis that the ability to make and use sharp stone tools may have developed in our own lineage after our split with chimpanzees, approx. seven million years ago.

## Introduction

Sharp-edged flakes (henceforth flakes) played a key role in human evolution by allowing the exploitation of new ecological niches. The two earliest types of archaeological assemblages containing flakes are the Lomekwian (at 3.3Ma; Harmand *et al.*, 2015; but see Archer *et al.*, 2020; Domínguez-Rodrigo & Alcalá, 2016 for a debate on the Lomekwian contexts) and the Oldowan (at 2.58Ma; Braun *et al.*, 2019). Although it is widely accepted that intentional flake manufacture was a major milestone in hominin evolution ( Roche, 2000; Semaw *et al.*, 1997), it remains debated how this behaviour emerged and why reliable archaeological evidence is absent in the approximately four million years following the split between *Homo* and *Pan*. It has been suggested that the ability to manufacture flakes was acquired by naïve individuals via special mechanisms of cultural transmission, namely copying variants of social learning ( Stout *et al.*, 2019). Copying variants of social learning, such as imitation and some types of emulation, allow for the direct transmission of behavioural forms via the observation of a model or its products ( Bandini *et al.*, 2020; Tennie *et al.*, 2020b). However, the hypothesis that flake manufacture and use (especially in early stone artefact assemblages) were learned via copying is still debated ( Boyd & Richerson, 2005; Corbey *et al.*, 2016; Foley, 1987; Tennie *et al.*, 2016; Tennie *et al.*, 2017). Due to the impossibility of directly testing the learning mechanisms underlying flake manufacture and use in early hominins, one must resort to indirect methods in order to reconstruct early hominin learning processes. One such method involves the application of cognitive cladistics to examine how our ancestors may have acquired their behaviors by testing our closest living relatives, non-human great apes ( Arroyo *et al.*, 2016; Carvalho & McGrew, 2012; Panger *et al.*, 2002; Wynn *et al.*, 2011).

So far, only three ape subjects – one orangutan ( *Pongo pymaeus*; 'Abang'; Wright, 1972) and two bonobos ( *Pan paniscus*, 'Kanzi' and 'Panbanisha'; Schick *et al.*, 1999; Toth *et al.*, 1993) – have been tested for their ability to learn how to make and use flakes (note that two other juvenile bonobos, Panbanisha’s sons, were reported to have also acquired flake making abilities after observing Kanzi and Panbanisha, see below). Wright (1972) provided a male orangutan (Abang) with a fixed flint core, a loose river pebble that could be used as a hammerstone and a baited puzzle box that could only be opened with a sharp tool (by cutting a rope lock). Wright implemented two experimental conditions, which included both demonstrations and tests. In the first experimental condition, Wright tested Abang's abilities to learn how to use a flake as a cutting tool to open the puzzle box. In the second condition, Wright tested the orangutan's abilities to make his own flakes and subsequently use them to cut open the rope lock. Given Abang's initial failure to use a flake as a cutting tool in the first experimental condition, Abang’s keeper tried to elicit flake use by "guiding his hand to cut the string" of the rope lock ( Wright, 1972). After a total of nine human demonstrations of how to use a flake as a cutting tool (one of which involved the abovementioned physical guidance), Abang used a human-made flake as a cutting tool to open the puzzle box. In the second experimental condition, after seven human demonstrations of how to make a flake using freehand percussion (i.e. a technique where a hand-held hammerstone is used to detach flakes from a bodily stabilized or hand-held core; Schick & Toth, 1994), Abang made four flakes in succession using a hand-held hammerstone to hit on the fixed core. Abang subsequently used one of the flakes he made himself to cut through the rope locking the puzzle box and obtain the food reward ( Wright, 1972).

Twenty years later, Toth, Schick and colleagues adapted the methodology employed by Wright to test bonobos' flake manufacture and using abilities (e.g., Schick *et al.*, 1999; Toth *et al.*, 1993). The language-trained and enculturated bonobo Kanzi was provided with a puzzle box that, similarly to Abang’s puzzle box, could only be opened using a sharp tool to cut a rope lock. As in the earlier study with Abang, Kanzi was also provided with human demonstrations on how to detach flakes from a core using freehand percussion. Following these demonstrations, Kanzi was provided with loose cores and hammerstones. Although physical guidance did not take place, the bonobo was encouraged to make flakes by placing stones in his hands. In addition to the puzzle box with the rope lock, Kanzi was also presented in later experiments with a second puzzle box designed to resemble a drum with a taut plastic/silicone cover. This drum box allowed Kanzi to obtain a food reward only after cutting through the plastic cover with a sharp object (e.g., Schick *et al.*, 1999; Toth *et al.*, 1993).

Kanzi started using human-made flakes to open the puzzle boxes almost immediately after the experiments began ( Toth *et al.*, 1993). Eventually, Kanzi also reliably made flakes himself and used them to open the puzzle boxes. To make flakes, Kanzi brought down a hand-held hammerstone against a core either held in the other hand, braced against the floor with a foot or a hand, or on the ground ( Toth *et al.*, 1993). A couple of months into testing, Kanzi innovated a flake manufacturing technique that had not been modeled for him. Kanzi’s own technique involved forcefully throwing loose cores onto hard surfaces (throwing technique; Toth *et al.*, 1993) or objects (directed-throwing technique; Toth *et al.*, 1993). Later, Kanzi's half-sister Panbanisha was reported to have learnt how to make and use flakes after being provided with human demonstrations of freehand percussion ( Savage-Rumbaugh & Fields, 2006). However, Panbanisha's learning process and knapping skills were not described in detail. Similarly, Panbanisha's two sons were also reported to have learnt flake manufacture and use after observing Kanzi and Panbanisha. However, neither their learning process nor their behaviours (i.e. which techniques they used and which puzzle boxes they opened) were reported ( Toth *et al.*, 2006).

Although these early ape studies were clearly innovative in their methods, there are several factors that limit the conclusions that can be drawn from them. Firstly, all of the tested apes were enculturated to a certain degree. Enculturation refers to the rearing conditions of apes "in a human cultural environment, with wide exposure to human artifacts and social/communicative interactions" ( Furlong *et al.*, 2008; see also Henrich & Tennie, 2017). Enculturation severely limits the ecological relevance of test subjects’ behavior and cognition, reducing in turn the external validity of findings such as the ones described above. This is because enculturation is known to allow apes to acquire behavioural and cognitive abilities that are beyond those of wild and/or unenculturated apes, such as copying social learning ( Buttelmann *et al.*, 2007). Given that wild apes do not have access to human enculturation, findings from enculturated apes are of limited value in phylogenetic inferences. A second limitation of these early ape flaking studies is that, prior to test, all apes were provided with demonstrations of how to make and use flakes. Therefore, neither the spontaneous nor the naturally developing abilities of apes to make and use flakes have ever been investigated in unenculturated individuals (but see positive findings of flake manufacture and use in untrained, unenculturated capuchin ( *Sapajus apella*) monkeys in a similar experimental paradigm; Westergaard & Suomi, 1994). Finally, although chimpanzees are one of our two closest living relatives (alongside bonobos) and despite them showing by far the most extensive tool-use repertoires of all apes in the wild (including some stone tool behaviors such as nut-cracking with stone hammers; Whiten *et al.*, 1999; Whiten *et al.*, 2001), chimpanzees have never been tested before in knapping experiments.

Investigating individual flake manufacture and using abilities in the absence of demonstrations using task-naïve, unenculturated apes would provide insight on whether these behaviours are within the cognitive reach of ecologically representative apes. More generally, if such apes were found to spontaneously make and use flakes it would add further empirical evidence that, in species with broad tool repertoires, flake manufacture and use does not require copying social learning (following demonstrations) and/or cognitive skills potentially installed during human enculturation (similar to what was reported for naïve, unenculturated capuchin monkeys; Westergaard & Suomi, 1994). Finding flake making and/or use in naïve, unenculturated chimpanzees would be compatible with a scenario where these skills were also present in the last common ancestor of *Homo* and *Pan* seven million years ago. However, if contrary to tutored and enculturated bonobos and orangutans (see above), untutored, unenculturated chimpanzees would not make or use flakes, this would suggest that these abilities are beyond the natural cognitive reach of ecologically-representative subjects of this species. This latter finding would support a scenario in which the provision of human demonstrations and/or enculturation may be a pre-requisite for the development of flake making and use in chimpanzees.

We tested the largest experimental sample included in an ape flaking experiment to date by assessing the individual abilities of 11 task-naïve, unenculturated chimpanzees to make and use flakes. We tested these subjects across several experimental conditions in which different amounts of social information were successively provided in order to examine the level and type of information required for chimpanzees to develop flake manufacture and/or use (compare Bandini *et al.*, 2020). We also self-replicated our findings by testing chimpanzees across two different populations (Table S1 in Extended Data). As in the case of the tutored, enculturated ape subjects included in previous flaking studies ( Toth *et al.*, 1993; Wright, 1972), our untutored, unenculturated subjects were provided with the necessary materials to make flakes (hammerstones and cores) as well as opportunities and motivation to use them (two baited puzzle boxes that afforded the use of sharp tools equivalent to those employed in previous ape flaking studies). We predicted that if flake manufacture and use were within the natural individual cognitive reach of apes, the chimpanzees in our study would make and use flakes.

## Methods

### Study design

We tested task-naïve, unenculturated chimpanzees across two institutions (a sanctuary and a zoo; N _total_=11). We aimed *a priori* to test all zoo-housed chimpanzees (n=9) and all sanctuary housed chimpanzees belonging to the "Escape artists" group (n=4). Chimpanzees were included in the study if they participated in all trials. However, once testing started, two zoo-housed chimpanzees were excluded from the study. One zoo-housed female chimpanzee was excluded from the study as she chose not to participate in the experiments by not entering the testing quarters when the testing materials were placed inside it. One zoo-housed male was excluded from the study as his rearing background included enculturation in a human cultural environment (he lived with humans until he reached adolescence). Each chimpanzee included in the study was individually tested in order to ensure that if any chimpanzee performed the target behavior(s) (flake manufacture or use), this would not render the other chimpanzees nearby unsuitable for further testing (given that potential observers could not be considered task-naïve anymore). Thus, our experimental design allowed us to confidently conclude that any occurrence of the target behaviors during testing must have been individually learned and not copied from (or elicited by) others. Furthermore, we tested chimpanzees in two populations in order to a) self-replicate our findings; b) slightly vary specifics of our methods in order to maximize the chances of occurrence of the target behaviors; and c) account for potential inter-group differences in housing conditions.

Subjects were housed at Chimfunshi Wildlife Orphanage and Kristiansand Zoo. Chimfunshi Wildlife Orphanage is located in Zambia, Africa (12° 23’ S, 29° 32’ E). Four chimpanzees (mean _age_=29.5, range _age_: 18–46, 2F & 2M; Table S1 in Extended Data) were tested individually at Chimfunshi between 9:00 and 16:00 in July 2016. During tests, one chimpanzee was called into the management area while the other chimpanzees remained in the outdoor enclosure. The chimpanzees had access to a seven-hectare outdoor enclosure and four indoor management areas. The outdoor enclosure was a fenced field with natural soil, rocks and vegetation. The chimpanzees spent the day primarily in the outdoor enclosure, and slept in the inside management rooms. Two daily feeds were provided between 11:30 and 12:30 and between 14:30 and 16:30. Kristiansand Zoo is located in Kristiansand, Norway. Seven chimpanzees (mean _age_=23.7, range _age_: 7–41, 4F & 3M; Table S1 in Extended Data) were tested individually at Kristiansand Zoo (except a female, Jane, and her dependent offspring, which were tested together) in their sleeping quarters during the morning cleaning routines between 7:30 and 8:30 in May, June and November 2018. During this time, each individual was kept in their sleeping quarter, separated from the other chimpanzees by brick walls that prevented visual contact between the chimpanzees. The chimpanzees at Kristiansand Zoo had access to two enclosures (one indoors and one outdoors), as well as to a separate indoor sleeping area where the tests took place. The indoor enclosure was equipped with several enrichment devices commonly found in zoological institutions (e.g., artificial termite mount, climbing frames). The outdoor enclosure was an island of 1840 m ^2^ surrounded by a water-filled moat, with natural soil, rocks and vegetation. The indoor sleeping area was off-exhibit. Two daily feeds were provided at 10:00 and 14:00. Food was also scattered at 9:30 in the indoor and outdoor enclosures. It was decided *a priori* that only chimpanzees that participated in all the test trials would be included in the study. All individuals entered the testing rooms voluntarily, and therefore could choose not to participate in the experiments.

**Figure 1.**
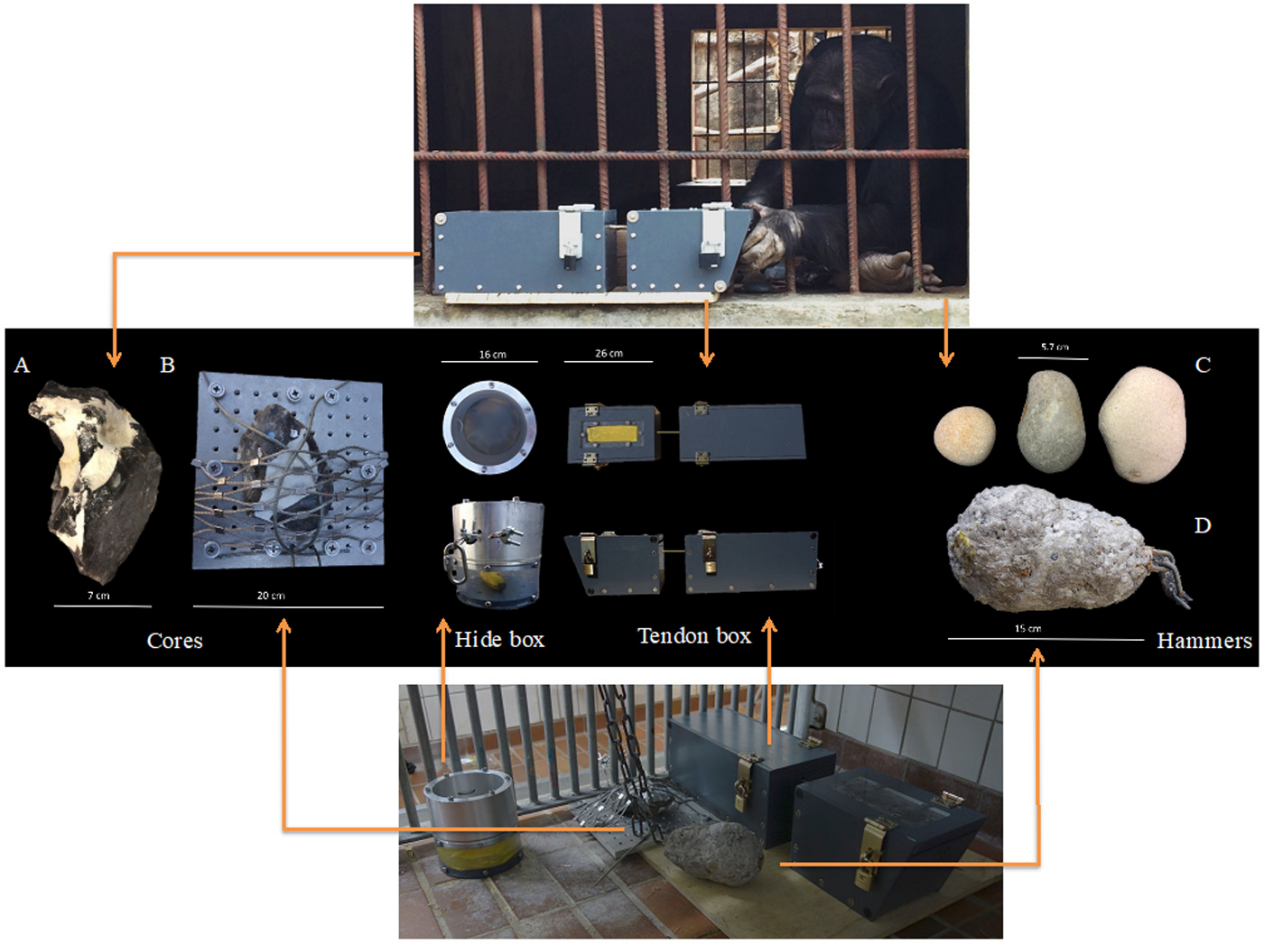
Experimental set-up. Testing materials used in Chimfunshi Wildlife Orphanage (core A, tendon box and hammers C) and Kristiansand Zoo (core B secured to the mesh of the enclosure, hide box, tendon box and hammer D). At Chimfunshi (top picture), individuals were provided with a loose core ( **A**), the baited "tendon box" (where a rope acted as a tendon substitute) and three loose hammerstones ( **C**). At Kristiansand Zoo (bottom picture), individuals were provided with a fixed core ( **B**), the baited "hide box", the baited tendon box and an artificial hammer ( **D**). Both boxes were modeled after those used in the previous ape flaking studies and the food rewards contained within could only be obtained using a cutting tool. The arrows link each chimpanzee population with the materials provided during the experiments (middle panel).

Two different puzzle boxes baited with food items were used in order to motivate the chimpanzees to make (and subsequently use) flakes. Both puzzle boxes used in this study were novel for all the tested chimpanzees. However, the chimpanzees at both institutions were familiar with test apparatuses in general. Indeed the provision of the puzzle boxes served as part of the enrichment routine practiced in the testing facilities. In both testing institutions we used a puzzle box with a rope lock inspired by previous flaking experiments with great apes (e.g., Schick *et al.*, 1999; Toth *et al.*, 1993; Wright, 1972) that we named the "tendon box" ( Figure 1). The tendon box was used to simulate a scenario in which, faced with an animal carcass, a subject must cut through taut tendons (a rope in our experiment) in order to dismember a body. Our tendon box consisted of two opaque boxes secured to a wooden board [box one (rewarded box): 26 × 17.3 × 17.3 cm; box two (non-rewarded): 36 × 15 × 17.2 cm]. The tendon box had a clear Plexiglas window (5 × 16 cm) at the top that allowed the reward inside to be visible to the chimpanzees. The door of the box was pulled shut by a rope that ran through the inside and exited through a hole in the opposite end. The rope then ran between the two boxes for approximately 5 cm and entered the second (non-rewarded) box. The rope was secured in the non-rewarded box to a clamp that could be tightened to ensure that the rope was taut. The rope was only accessible in the area between the two boxes, and had to be cut there to allow the door of box one to open. The rope was a brown twisted cord hemp rope, approx. 2 mm thick. This type of rope was selected as it was found to be (after pilot testing by EB) strong enough to withstand attempts by a human at opening the box without a tool but could be cut using a knife or flake. Collectively, the box weighed approximately 21 kg (including the board on which the boxes were fixed).

We also used a second puzzle box in Kristiansand Zoo named the “hide box”. The hide box design was inspired by the additional box used in the bonobo knapping experiments of Toth *et al.* (1993) as well as in the capuchin monkeys knapping experiments of Westergaard & Suomi (1994). This box roughly resembled a drum with an occluding silicone membrane 2 mm thick on top ( Figure 1) and consisted of a transparent Plexiglass cylinder (16 cm wide × 15.5 cm high) with a metallic rim. The silicone membrane was screwed in between the cylinder and the rim, blocking the access to the reward placed inside the cylinder. The hide box was then secured to the bars of the rooms where the experiments took place ( Figure 1).

The use of puzzle boxes baited with food is a common practice in cognitive experiments investigating animal's problem solving abilities (e.g., Buttelmann *et al.*, 2013; Forss *et al.*, 2020; Hopper *et al.*, 2007; Hopper *et al.*, 2008; Whiten *et al.*, 2005; Whiten *et al.*, 2007). The rewards (baits) placed inside our two puzzle boxes included in our experiments consisted of peanuts and animal biscuits in Chimfunshi and half a banana or a yogurt in Kristiansand. The rewards were chosen based on the advice provided by the keepers regarding which were the preferred foods of the chimpanzees at each site.

In Chimfunshi, the chimpanzees were provided with three oval loose hammerstones (small, medium and large) in each trial (weight range 0.5–1 kg). Hammerstones were collected from streams around Birmingham, UK, based on the size and shape (similar to a potato) of the ones most commonly found in archaeological assemblages ( Mora & De la Torre, 2005). Due to safety regulations, it was not possible to provide loose hammerstones to the chimpanzees housed at Kristiansand Zoo. Instead, one concrete rounded hammer (ca. 15 cm long × 10 cm wide, weight 2.2 kg) was provided during each trial. The weight of the hammer was modeled based on the hammers used by wild chimpanzees to crack nuts ( Biro *et al.*, 2003). The hammer was built around a metallic scaffold linked to a chain that allowed us to fix it to the bars of the testing rooms so the chimpanzees could not carry the hammer into the indoor enclosure. The concrete used to make the hammer included particles of up to 1 cm in diameter ( Figure 1). The hammer was covered with non-toxic transparent epoxy resin to prevent its surface from disintegrating upon hammering.

In both sites, retouched Norfolk Chert cores were provided to the chimpanzees alongside the hammers ( Figure 1). Unworked cores were purchased from a provider (Needham Chalks) in the UK and then knapped at the University of Birmingham. The cores were partially decortified to make the actual flint accessible, and in order to create platform angle variability between ~90 degrees and ~30–40 degrees which would make flake removal possible at the outset (similar to the procedure used in the earlier knapping studies; Toth *et al.*, 1993; Westergaard & Suomi, 1994; Wright, 1972). During this process we aimed to produce either i) three separate surfaces with varying angles from which flakes could potentially be struck or ii) a continuous edge around the perimeter of the core with continuously varying angles within the above mentioned platform angle range. The cores weighed between 0.8 and 1.5 kg. Subjects received one core per trial and if the core was not modified, the core was used in further trials. In Chimfunshi, cores were provided loose to the chimpanzees and therefore could have been reduced with various techniques. Due to safety regulations, in Kristiansand Zoo the core had to be fixed on a metallic platform (20 × 20 × 2 cm) to prevent the chimpanzees from carrying the core into the indoor enclosure. Similarly to the previous orangutan experiment ( Wright, 1972), the stationary position of the core limited the possible techniques of flake removal. The core was attached to the metallic platform using a metallic wired mesh with openings 5 cm wide and 3 mm wire (XTEND, Carl Stahl ARC GmbH, Architectural Cables and Mesh Systems), leaving a knappable section of the core (with a platform angle of less than 90 degrees) exposed.

In Chimfunshi, the tendon box was set up on a ledge outside of the testing area and baited before the subjects entered the room. This set up was chosen to increase the visibility of the tendon box from the experimenter’s location 3 m away from the room's bars. Three hammerstones and one core were placed – all unfixed – on the floor inside the enclosure, allowing the chimpanzees to freely manipulate them. One camera (Sony HDR-CX330E Handycam) was set-up one meter from the enclosure, and recordings started once a subject entered the testing area. In Kristiansand, all testing materials (hide and tendon boxes, the artificial hammer and the fixed core) were placed inside the testing room and secured to the bars of the enclosure. All materials had to be placed inside the testing room because the chimpanzees could not extend their arms through the bars (as in Chimfunshi) due to safety reasons. Two Sony HDR-CX330E Handycams were set-up half a meter from these bars, and started recording once the subject entered the testing room. Potential tools were cleared from the testing areas before the tests started. However, the chimpanzees often brought tools with them into the testing areas at the start of the tests.

### Experimental design

We implemented two experimental conditions: a baseline and a flake condition. During the baseline the subjects were provided with the testing materials but no demonstrations, guidance, or artefacts (e.g., no pre-made flakes) were provided. Crucially, no information regarding how to manufacture or use flakes was given before or during this condition. The aim of the baseline condition was to investigate whether chimpanzees could individually learn flake manufacture and use (as they were required to make a flake before they could use it). In Kristiansand Zoo, the baseline condition was split into two other sub-conditions (baseline condition I and baseline condition II) to control for the potential effect of testing with two baited boxes instead of one (as in Chimfunshi). During the baseline condition I, seven chimpanzees were provided with the tendon box, the hide box, a hammer and a fixed core ( Figure 1). All chimpanzees in Kristiansand Zoo were tested individually in three trials each during the baseline condition I (condition duration range 01:05:40 to 03:00:49). We included a second baseline in Kristiansand Zoo to focus the attention of the individuals on solving a single task by only providing them with one box. In the baseline condition II, only the four most engaged individuals (two males and two females) of the seven that participated in the baseline condition I were tested. The box that each individual was tested with (tendon or hide box) was assigned randomly. These four individuals were tested in three additional trials each during the baseline condition II (condition duration range 01:18:50 to 03:31:12). The individual trial length varied (range 00:29:14 to 02:02:19) depending on the duration of the local cleaning routines, as this was the time when testing took place. The same four individuals that we tested in the baseline condition II were further tested in the flake condition (condition duration range 01:46:23 to 02:38:32).

In the flake condition we used the same materials as in the baseline condition but we also provided the chimpanzees with a pre-made flake. The aim of the flake condition was to test if the chimpanzees could spontaneously recognize a flake as a potential cutting tool to access the puzzle boxes. The flake provided during this condition was made out of chimpanzees’ sight by the experimenters using freehand percussion. In Chimfunshi the flake measures were: platform depth = 8.46 mm, platform width = 21.46 mm, technological length = 50.76 mm and flake width = 47.56 mm. In Kristiansand the flake measures were: platform depth = 10.93 mm, platform width = 25.9 mm, technological length = 61.73 mm and flake width = 42.36 mm. Platform depth was measured as the distance from the impact point along the platform surface to the exterior margin of the flake and perpendicular to the interior surface of the flake. Platform width was measured from one lateral margin of the platform to the other. Flake length was measured from the impact point to the most distal point of the flake and flake width as the distance between the two flake edges at the midpoint and perpendicular to the length axis. Before the start of the flake condition, the experimenters tested the functionality of the flakes by opening the puzzle boxes themselves (only flakes that could cut open the puzzle box were provided). Each flake was placed unfixed (loosely on the floor) next to the hammerstone(s), core and puzzle box(es) before the subjects were allowed into the testing rooms.

All four chimpanzees at Chimfunshi were tested in the baseline condition II setup and the flake condition. Each trial lasted 20 minutes. The four chimpanzees at Chimfunshi were tested in three trials of the baseline condition II (60 min in total per individual) and in two trials of the flake condition (40 min in total per individual).

### Coding

From each video-recorded trial we coded i) the duration of the interactions (time spent in physical contact with the testing materials, from when the subject started contact until it paused for more than 3s or changed activity), ii) which testing material the chimpanzees interacted with and iii) if the interaction was manual or using a tool.

### Flake data capture

The two flakes provided during the flake condition were scanned with an Artec Space Spider 3D scanner using the data capture software Artec Studio 14 (Figure S1 in Extended Data). Similar scans could be created using photogrammetric approaches with freely available software like VisualSFM ( Wu, 2011).

### Statistical analysis

A proportion (20%) of the interactions between the chimpanzees and the testing materials across experimental conditions from each institution were re-analysed by a second coder naïve to the goals of the experiment in order to assess the inter-rater reliability. The second coders (AC & LK) were asked to re-analyse the videos based on a provided ethogram (Table S2 in Extended Data). The clips of the interactions provided to the second coders were randomly selected using a number generator and a number of dummy clips, where no interaction took place (10% of the total number of interactions), were included as a control. The second coders' analysis was compared to the original analysis using Cohen's Kappa statistic. No statistical comparisons between individuals of the two housing facilities or experimental conditions were conducted.

To determine whether our sample size was suitably powered to test for the ability to manufacture and use flakes, the probability of the chimpanzees in our study *not* performing the target behaviors was calculated from a binomial probability distribution using the function dbinom from the R software version 3.6.1 (2019-07-05). The expected probability of the behaviors in the population was obtained from the only previous study that tested the spontaneous flake making abilities of naive, unenculturated primates ( Westergaard & Suomi, 1994), in which 60% of the individuals (9 out of 15) spontaneously made flakes from a provided core. For our power analysis (see Figure S2 in Extended Data) we used the incidence of flake making and use in naïve, unenculturated capuchin monkeys ( Westergaard & Suomi, 1994) as the probability that naïve, unenculturated chimpanzees would also innovate this behavior. If we had based our analysis instead on the previous incidence of flake making and using of enculturated and trained apes, the expected probability of the behavior would have been 100% (based on the results of Toth *et al.*, 1993; Wright, 1972).

### Ethics

The experiments reported comply with the Guide for the Care and Use of Laboratory Animals ( National Research Council, 2011), the American Society of Primatologists’ Principles for the Ethical Treatment of Primates, and with current Norwegian laws. The experiments were approved by the Ethical commission of the European Research Council (ERC). This study was further approved by the ethical board of Kristiansand Zoo before its commencement. The research at Chimfunshi was approved by the University of Birmingham research board (reference UOB 31213), in line with the requirements for testing of animals in the UK and internationally. The project was also approved by CRAB (Chimfunshi Research Advisory Board). All participation in the study was voluntary. The subjects were called by name into the testing quarters and could choose not to enter the testing rooms, and thus not participate in the study. If the subject chose to enter the testing quarters and thus participate in the study, the chimpanzees were free to interact as much or as little as they wanted with the testing materials. The experimenters never attempted to encourage the chimpanzees to interact or manipulate the testing materials, but merely observed their behavior during the trials. If the subjects showed any signs of distress or tried to exit the testing room by manipulating the door during testing, they would immediately be released back into their main enclosure. Subjects were never food or water-deprived, and continued with their regular feeding routine during the study. Subjects had access to water *ad libitum* prior *,* during, and after testing. The chimpanzees included in the study were used to being separated from their group for short periods of time during cleaning routines or veterinary check-ups. Therefore, the keepers and the research boards of both testing institutions agreed that the separation of the chimpanzees for this study would not cause any harm or distress to the chimpanzees. Nevertheless, the experimenters were always present during the experiments (alongside one chimpanzee keeper) and would have terminated the trial immediately if the chimpanzees had shown any signs of distress (this never occurred during the present study). No incidents or adverse events occurred during data collection for the present study.

## Results

There was a substantial to almost perfect agreement ( Cohen, 1988) between the two coders of the interactions between the chimpanzees and the testing materials at Chimfunshi (k=0.684) and Kristiansand Zoo (k=0.947) ( Motes-Rodrigo & Bandini, 2021). Regarding the chimpanzee's spontaneous knapping abilities, none of the chimpanzees included in our sample made flakes in either the baselines (when no information or final products were provided) nor flake condition (when a human-made flake was provided). In addition, no chimpanzee used the provided flake during the flake condition to open any of the baited boxes.

Modeled on the results from the only previous study that tested the spontaneous flake making abilities of naïve, unenculturated primates ( Westergaard & Suomi, 1994), we found that the probability that we would not find this behavior even once in our ape sample was 0.0004. Such low probability indicates that the probability that chimpanzees would be as proficient as capuchins in their stone tool making and using abilities is extremely low (Figure S2 in Extended Data).

Although the chimpanzees in our sample did not make or use flakes, they interacted frequently with the testing materials, thus proving motivated to retrieve the food rewards from inside the puzzle boxes (Table S3 in Extended Data). In Chimfunshi, the total interaction time with the testing materials was 00:54:37, while at Kristiansand Zoo it was 02:05:21. These differences between sites are likely due to the longer trials and the larger number of individuals tested at Kristiansand Zoo compared to Chimfunshi. In Chimfunshi, the chimpanzees interacted the most with the tendon box (00:50:34) and the least with the flake (in the flake condition; 00:00:25). In Kristiansand Zoo the chimpanzees interacted the most (01:35:18) with the hide box (the hide box was not available at Chimfunshi) and the least with the flake (in the flake condition; 00:00:08). In Kristiansand Zoo, the chimpanzees interacted with the tendon box for a total of 00:12:53. Interactions were made both by hand and using tools (Table S4 in Extended Data). In both institutions, the chimpanzees frequently interacted with the door and rope lock of the tendon box and with the membrane of the hide box in Kristiansand, suggesting that they understood the opening mechanisms of the boxes. The chimpanzees used straws, plastic hose fragments, plastic cups, sticks and plastic pieces that they retrieved on their own as tools. However, the chimpanzees were never successful in opening the boxes using any of these tools. Chimpanzees at both institutions knocked (touched repeatedly and in quick succession an object with the knuckles), slapped (touched in a fast movement an object with the palm of the hand) and hit (touched fast and using considerable force an object with any part of hand other than the palm) the testing materials provided. However, no percussive actions with a tool (e.g. hammers) took place in any of the trials.

## Discussion

In contrast to the earlier ape flaking studies using tutored, enculturated apes, none of the 11 untutored, unenculturated chimpanzees we tested made or used a flake neither during the baseline conditions nor after being given a pre-made functional flake (flake condition). Such negative results occurred even though the chimpanzees understood the task requirements and were motivated to open the puzzle boxes. It is unlikely that the notable absence of flake production and use in our study as compared to previous ape flaking studies is due to inter-species differences in cognitive and/or physical abilities. Cognitively, chimpanzees are at least on par in physical skills with orangutans and bonobos as they show by far the most extensive tool-use repertoires of all wild apes (which includes lithic percussive behaviours; Whiten *et al.*, 1999; Whiten *et al.*, 2001). Chimpanzees are also physically able to produce flakes, as evidenced by several reports of wild chimpanzees unintentionally detaching flake-like objects while engaging in nut-cracking using stone hammers and anvils ( Carvalho *et al.*, 2008; Mercader *et al.*, 2007; Mercader *et al.*, 2002).

A more likely explanation for the discrepancy between the results of our study and those of previous ape flaking studies is the background and experiences of the subjects, both in a long timeframe and immediately before testing took place. Contrary to the apes tested in the early flaking experiments, the chimpanzees included in our study were not enculturated nor had been exposed to extensive human training. Furthermore, the chimpanzees in our study were untutored in the target behaviours as they were not provided with social demonstrations of how to make or use flakes, whereas all previously tested apes ( Toth *et al.*, 1993; Wright, 1972) were exposed to human demonstrations before the onset of the experiments. It is therefore likely that enculturation had a large role in driving the findings of earlier reports of ape flake making and use. Not only do enculturated apes generally show skills and cognitive abilities different from unenculturated apes (e.g., Tomasello & Call, 1997), but previous studies have shown that enculturation and/or extensive human training in certain tasks (e.g. Pope *et al.*, 2018) predisposes apes to attend to and even in some cases copy behavioural forms ( Buttelmann *et al.*, 2007; Tomasello *et al.*, 1993). That is, the degree of human enculturation of the previously tested apes would have predisposed them to attend and perhaps reproduce the human demonstrations of flake making and use provided in these earlier studies ( Toth *et al.*, 1993; Wright, 1972). As for the provision of demonstrations in earlier ape flaking experiments, it is unlikely that demonstrations alone could have elicited flake making and use considering that unenculturated apes do not spontaneously copy behaviour ( Clay & Tennie, 2017; Tennie *et al.*, 2012) or artefact forms ( Tennie *et al.*, 2009) that they could not produce on their own in baseline conditions (see also Tennie *et al.*, 2020a). Consequently, previous studies suggest that unenculturated apes (including the chimpanzees tested in our study) are unlikely to make and use flakes following human demonstrations if these behaviours are not already expressed in baseline conditions. Although studies testing the individual contributions of enculturation and demonstrations to the expression of flake manufacture and use abilities in apes are still pending, the available evidence suggests that the previously tested orangutan and bonobo learnt how to make and use flakes because their enculturated state predisposed them to attend and reproduce social demonstrations.

Our results suggest that, outside the sphere of human enculturation (in combination with human demonstration and/or molding), the individual abilities of chimpanzees (and by phylogenetic proxy, that of hominins with chimpanzee-like cognitive abilities), do not seem sufficient to manufacture or use flakes. Assuming this interpretation is correct, there exist several possible evolutionary scenarios for the development of flake manufacture and use abilities in the hominid lineage. The first possible scenario is that hominin species pre-dating both the last common ancestor of chimpanzees and humans, as well as hominins with chimpanzee-like cognitive abilities, were able to intentionally manufacture and use flakes, but this ability was subsequently lost in the *Pan* lineage, and maintained in the hominin lineage. If this scenario did indeed occur, we would expect that hominin species that evolved after the hominin split from *Pan* (approximately seven million years ago) would have engaged in flake manufacture and use. However, there is a distinct absence of flaked stone tools in the archaeological record for millions of years after the split between hominins and the genus *Pan*. This gap in the archaeological record could be due to a very low density of manufactured flakes in the environment, which in addition to their archaic characteristics, would render their identification in archaeological excavations difficult ( Semaw *et al.*, 1997). Low flake manufacture densities during this period could be explained by a lack of necessity for flakes in the specific ecological niches inhabited by the different hominin species. An alternative scenario would be one in which hominoids with equivalent cognitive abilities to chimpanzees did not have the ability to make flakes. According to this scenario, the ability to manufacture flakes would have evolved later in the hominin lineage (and then may or may not have remained dormant), resulting in certain hominin species eventually crossing the cognitive Rubicon for flake manufacture and use. In both of these scenarios, it remains an open question whether learning mechanisms involved in modern human cultural transmission (especially copying social learning mechanisms; Tennie *et al.*, 2017) were responsible for the acquisition of flake manufacture and/or use abilities.

Flake manufacture and use independent of copying social learning has already been suggested to be the most likely explanation for a phylogenetically independent case of flake manufacture and use in naïve (G. Westergaard, pers. comm), unenculturated capuchin monkeys ( Westergaard & Suomi, 1994). Two previous studies tested the spontaneous flaking abilities of capuchins monkeys ( *Sapajus apella*; Westergaard & Suomi, 1994; Westergaard & Suomi, 1995). To examine the spontaneous flaking abilities of this species, Westergaard and Suomi implemented a similar methodology to our baseline condition by providing task-naïve, unenculturated capuchins with the necessary materials (hammers and cores) and motivation (puzzle box baited with food similar to the hide box) to make and use flakes ( Westergaard & Suomi, 1994). In contrast to the chimpanzees tested in our study, the capuchins spontaneously made and used flakes in independent baseline conditions, thus validating the testing paradigm used in our study. As the tested capuchins were naïve to flake manufacture and use prior to and during testing (i.e. no demonstrations were provided) this capuchin study represents a proof of principle that the ability to manufacture and use flakes as cutting tools in at least some species of tool-using primates can develop in the absence of copying opportunities and enculturation ( Westergaard & Suomi, 1994).

The present study strongly suggests that contrary to the capuchins, naïve unenculturated chimpanzees do not possess or develop flake making and using abilities spontaneously. Such inter-species differences could perhaps be explained by different genetic predispositions for stone manipulation in capuchins and chimpanzees ( Hayashi, 2015). Taken together, the results of this study, the capuchin data and the archaeological record, support a scenario in which the abilities to make and use flakes would have evolved independently, and at least twice, during primate evolution: in capuchins (see also Proffitt *et al.*, 2016 for flake production in wild capuchins) and at least once in the hominin lineage once cognitive abilities more advanced than those found in chimpanzees developed and/or the ecological pressure for sharp tools emerged. This scenario would also explain the large time gap (spanning several million years) between the split of hominins and *Pan* and when the first flaked stone tools appear in the archaeological record (currently circa 2.58 million years: Braun *et al.*, 2019).

## Data availability

### Underlying data

Open Science Framework: Naïve, unenculturated chimpanzees fail to make and use flaked stone tools. https://doi.org/10.17605/OSF.IO/5UWKA ( Motes-Rodrigo & Bandini, 2021).

This project contains the following underlying data:

-  coding stonecult AMR kristiansand.csv (Data coded from the experiments conducted at Kristiansand Zoo)

-  coding stonecult EB chimfunshi.csv (Data coded from the experiments conducted at Chimfunshi Wildlife Orphanage)

-  second_coder_data_louise.csv (Data coded by the second coder from Kristiansand Zoo)

-  second_coder_data_MB.xlsx (Data coded by the second coder from Chimfunshi Wildlife Orphanage)

### Extended data

Open Science Framework: Naïve, unenculturated chimpanzees fail to make and use flaked stone tools. https://doi.org/10.17605/OSF.IO/5UWKA ( Motes-Rodrigo & Bandini, 2021).

This project contains the following extended data:

-  Dictionary variable names.docx

-  Extended data figures and tables.docx

-  Power simulations Bandini, Motes-Rodrigo *et al.* R

-  stonecult_SubKnerten_ConFlaketendon_s23_t44_Cam2.mp4

-  stonecult_SubJosefine_ConFlakedrum_s22_t42_Cam1.mov

-  Chimfunshi_SubChiffon_ConBaseline.MPEG

Data are available under the terms of the Creative Commons Attribution 4.0 International license (CC-BY 4.0).
