# Naïve, unenculturated chimpanzees fail to make and use flaked stone tools [version 2; peer review: 3 approved]

**DOI:** 10.12688/openreseurope.13186.1

**Published:** 2021-07-15

**Authors:** Elisa Bandini, Alba Motes-Rodrigo, William Archer, Tanya Minchin, Helene Axelsen, Raquel Adriana Hernandez-Aguilar, Shannon P. McPherron, Claudio Tennie

**Affiliations:** 1 Department of Early Prehistory and Quaternary Ecology, University of Tübingen, Tübingen, 72070, Germany; 2 Department of Human Evolution, Max Planck Institute for Evolutionary Anthropology, Leipzig, 04103, Germany; 3 Kristiansand Zoo, Kardemomme By, Kristiansand, 4609, Norway; 4 Department of Social Psychology and Quantitative Psychology, University of Barcelona, Serra Hunter Program, Barcelona, 08035, Spain; 5 Centre for Ecological and Evolutionary Synthesis, University of Oslo, Oslo, NO-0316, Norway

**Keywords:** chimpanzee flaking, chimpanzee tool use, lithic technologies, hominid material culture

## Abstract

**Background:**

Despite substantial research on early hominin lithic technologies, the learning mechanisms underlying flake manufacture and use are contested. To draw phylogenetic inferences on the potential cognitive processes underlying the acquisition of both of these abilities in early hominins, we investigated if and how one of our closest living relatives, chimpanzees (*Pan troglodytes*), could learn to make and use flakes.

**Methods:**

Across several experimental conditions, we tested eleven task-naïve chimpanzees (unenculturated n=8, unknown status n=3) from two independent populations for their abilities to spontaneously make and subsequently use flakes as well as to use flakes made by a human experimenter.

**Results:**

Despite the fact that the chimpanzees seemed to understand the requirements of the task, were sufficiently motivated and had ample opportunities to develop the target behaviours, none of the chimpanzees tested made or used flakes in any of the experimental conditions.

**Conclusions:**

These results differ from all previous ape flaking experiments, which found flake manufacture and use in bonobos and one orangutan. However, these earlier studies tested human-enculturated apes and provided test subjects with flake making and using demonstrations. The contrast between these earlier positive findings and our negative findings (despite using a much larger sample size) suggests that enculturation and/or demonstrations may be necessary for chimpanzees to acquire these abilities. The data obtained in this study are consistent with the hypothesis that flake manufacture and use might have evolved in the hominin lineage after the split between *Homo* and *Pan* 7 million years ago, a scenario further supported by the initial lack of flaked stone tools in the archaeological record after this split. We discuss possible evolutionary scenarios for flake manufacture and use in both non-hominin and hominin lineages.

## Introduction

Sharp-edged flakes (henceforth flakes) played a key role in human evolution by allowing the exploitation of new ecological niches. The two earliest types of archaeological assemblages containing flakes are the Lomekwian (at 3.3Ma; Harmand et al., 2015; but see Archer et al., 2020; Domínguez-Rodrigo & Alcalá, 2016 for a debate on the Lomekwian contexts) and the Oldowan (at 2.58Ma; Braun et al., 2019). Although it is widely accepted that intentional flake manufacture was a major milestone in hominin evolution (Roche, 2000; Semaw et al., 1997), it remains debated how this behaviour emerged and why reliable archaeological evidence is absent in the approximately four million years following the split between *Homo* and *Pan*. It has been suggested that the *know-how* required for flake manufacture was acquired by naïve individuals via special mechanisms of cultural transmission, namely copying variants of social learning (Stout et al., 2019). Copying variants of social learning, like imitation and some types of emulation (such as end-state emulation, Byrne & Russon, 1998) allow for the direct transmission of behavioural forms (encompassing bodily actions and/or artefacts) via the observation of a model or its products (Bandini et al., 2020; Tennie et al., 2020b). However, the hypothesis that flake manufacture and use (especially in early stone artefact assemblages) were learned via copying is still debated (Boyd & Richerson, 2005; Corbey et al., 2016; Foley, 1987; Tennie et al., 2016; Tennie et al., 2017). Due to the impossibility of directly testing the learning mechanisms underlying flake manufacture and use in early hominins, one must resort to indirect methods in order to reconstruct early hominin learning processes. One such method involves the application of cognitive cladistics to examine how our ancestors may have acquired their behaviors by testing our closest living relatives, non-human great apes (Arroyo et al., 2016; Carvalho & McGrew, 2012; Panger et al., 2002; Wynn et al., 2011).

So far, only three ape subjects – one orangutan (*Pongo pygmaeus*; ‘Abang’; Wright, 1972) and two bonobos (*Pan paniscus*, ‘Kanzi’ and ‘Panbanisha’; Schick et al., 1999; Toth et al., 1993) – have been tested for their ability to learn how to make and use flakes (note that two other juvenile bonobos, Panbanisha’s sons, were reported to have also acquired flake making abilities after observing Kanzi and Panbanisha, see below). Wright (1972) provided a male orangutan (Abang) with a fixed flint core, a loose river pebble that could be used as a hammerstone and a baited puzzle box that could only be opened with a sharp tool (by cutting a rope lock). Wright implemented two experimental conditions that included both demonstrations and tests. In the first experimental condition, Wright tested Abang’s abilities to learn how to use a flake as a cutting tool to open the puzzle box. In the second condition, Wright tested the orangutan’s abilities to make his own flakes and subsequently use them to open the puzzle box. Given Abang’s initial failure to use a flake as a cutting tool in the first experimental condition, Abang’s keeper tried to elicit flake use by “guiding his hand to cut the string” of the rope lock (Wright, 1972). After a total of nine human demonstrations of how to use a flake as a cutting tool (one of which involved the above-mentioned molding), Abang used a human-made flake as a cutting tool to open the puzzle box. In the second experimental condition, after seven human demonstrations of how to make a flake using freehand percussion (i.e. a technique where a hand-held hammerstone is used to detach flakes from a bodily stabilized or hand-held core; Schick & Toth, 1994), Abang made four flakes in succession using a hand-held hammerstone to hit on the fixed core. Abang subsequently used one of the flakes he made himself to cut through the rope locking the puzzle box and obtain the food reward (Wright, 1972).

Twenty years later, Toth, Schick and colleagues adapted the methodology employed by Wright to test bonobos’ flake manufacture and using abilities (e.g., Schick et al., 1999; Toth et al., 1993). The language-trained and enculturated bonobo Kanzi was provided with a puzzle box that, similarly to Abang’s puzzle box, could only be opened using a sharp tool to cut a rope lock. As in the earlier study with Abang, Kanzi was also provided with human demonstrations of how to detach flakes from a core using freehand percussion. Following these demonstrations, Kanzi was provided with loose cores and hammerstones. Although molding did not take place in this case (to the best of our knowledge), the bonobo was encouraged to make flakes by placing stones in his hands. In addition to the puzzle box with the rope lock, Kanzi was also presented in later experiments with a second puzzle box designed to resemble a drum with a taut plastic/silicone cover. This drum box allowed Kanzi to obtain a food reward after cutting through the artificial cover with a sharp object (e.g., Schick et al., 1999; Toth et al., 1993).

Kanzi started using human-made flakes to open the puzzle boxes almost immediately after the experiments began (Toth et al., 1993). Eventually, Kanzi also reliably made flakes himself and used them to open the puzzle boxes (but see Eren et al., 2020). To make flakes, Kanzi brought down a hand-held hammerstone against a core either held in the other hand, braced against the floor with a foot or a hand, or on the ground (Toth et al., 1993). A couple of months into testing, Kanzi innovated a flake manufacturing technique that had not been modeled for him. Kanzi’s own technique involved forcefully throwing loose cores onto hard surfaces (throwing technique; Toth et al., 1993) or objects (directed-throwing technique; Toth et al., 1993). Later, Kanzi’s half-sister Panbanisha (who was also enculturated), was reported to have learnt how to make and use flakes after being provided with human demonstrations of freehand percussion (Savage-Rumbaugh & Fields, 2006). However, Panbanisha’s learning process and knapping skills were not described in detail. Similarly, Panbanisha’s two sons were also reported to have learnt flake manufacture and use after observing Kanzi and Panbanisha. However, neither their learning process nor their behaviours (i.e. which techniques they used and which puzzle boxes they opened) were reported (Toth et al., 2006).

Although these early ape studies were clearly innovative in their methods, there are several factors that limit the conclusions that can be drawn from their results. Firstly, all of the tested apes were enculturated at least to a certain degree. Enculturation refers to the rearing conditions of apes “in a human cultural environment, with wide exposure to human artifacts and social/communicative interactions” (Furlong et al., 2008; see also Henrich & Tennie, 2017). Enculturation severely limits the ecological relevance of apes’ behavior and cognition, reducing in turn the external validity of findings such as the ones described above. This is because enculturation and extensive training are known to change apes’ brain connectivity (Pope et al., 2018) as well as allow apes to acquire innovative and cognitive abilities that are beyond those of wild and/or unenculturated apes (e.g. copying social learning: Buttelmann et al., 2007; secondary representation: Suddendorf & Whiten, 2001; see also Tennie, 2019). For example, when testing the abilities of enculturated and semi-enculturated chimpanzees to correctly choose a functional raking tool, Furlong et al. (2008) found that enculturated apes outperformed both semi-enculturated and unenculturated conspecifics. Thus, given that wild apes do not have access to human enculturation, findings from enculturated apes are of limited value in phylogenetic inferences.

A second limitation of these early ape flaking studies is that, prior to test, all apes were provided with demonstrations of how to make and use flakes. Consequently, neither the spontaneous nor the naturally developing abilities of apes to make and use flakes have ever been investigated, as this would require testing unenculturated individuals in the absence of demonstrations (as has been previously done with untrained and unenculturated capuchin monkeys (*Sapajus apella*); Westergaard & Suomi, 1994). Finally, although chimpanzees are one of our two closest living relatives (alongside bonobos) and despite them showing by far the most extensive tool-use repertoires of all apes in the wild (including some stone tool behaviors such as nut-cracking with stone hammers; Whiten et al., 1999; Whiten et al., 2001), chimpanzees have never been tested before in knapping experiments.

Investigating individual flake manufacture and using abilities in the absence of demonstrations using task-naïve, unenculturated apes would provide insight on whether these behaviours are within the cognitive reach of ecologically representative apes. More generally, if such apes were found to spontaneously make and use flakes it would add further empirical evidence that, in species with broad tool repertoires, flake manufacture and use does not require copying social learning (following demonstrations) and/or cognitive skills potentially installed during human enculturation (similar to what was reported for naïve, unenculturated capuchin monkeys; Westergaard & Suomi, 1994). Finding flake making and/or use in naïve, unenculturated chimpanzees would be compatible with a scenario where these skills were also present in the last common ancestor of *Homo* and *Pan* approximately seven million years ago. However, if contrary to tutored and enculturated bonobos and orangutans (see above), untutored, unenculturated chimpanzees would not make or use flakes, this would suggest that these abilities are beyond the natural cognitive reach of ecologically-representative subjects of this species. This latter finding would support a scenario in which the provision of human demonstrations and/or enculturation may be a pre-requisite for the development of flake making and use in chimpanzees.

We tested the largest experimental sample included in an ape flaking experiment to date by assessing the individual abilities of 11 task-naïve, untrained chimpanzees to make and use flakes. Eight of the chimpanzees tested were mother-reared (unenculturated) but the enculturation status of the remaining three subjects is unknown. We tested these subjects across several experimental conditions in which different amounts of social information were successively provided in order to examine the level and type of information required for chimpanzees to develop flake manufacture and/or use (compare Bandini et al., 2020). We also self-replicated our findings by testing chimpanzees across two different populations (Table S1 in Extended Data). As in the case of the tutored, enculturated ape subjects included in previous flaking studies (Toth et al., 1993; Wright, 1972), our subjects were provided with the necessary materials to make flakes (hammerstones and cores) as well as opportunities and a motivation to use them (two baited puzzle boxes that afforded the use of sharp tools equivalent to those employed in previous ape flaking studies). In contrast to these earlier studies, we did not precede tests by demonstrations or molding. We predicted that if flake manufacture and use were within the natural individual cognitive reach of apes, the chimpanzees in our study would spontaneously make and use flakes.

## Methods

### Study design

We tested task-naïve, untrained chimpanzees across two institutions (a sanctuary; Chimfunshi Wildlife Orphanage, and a zoo; Kristiansand zoo; N_total_=11). We aimed *a priori* to test all nine zoo-housed chimpanzees and all sanctuary housed chimpanzees belonging to the “Escape artists” group (n=4). However, two zoo-housed chimpanzees were excluded from the study. One zoo-housed female chimpanzee was excluded from the study after testing started as she chose not to participate in the experiments by not entering her testing quarter when the testing materials were placed inside it. One zoo-housed male was excluded from the study before the start of the tests as his rearing background included potential enculturation in a human cultural environment (he lived with humans for a period of time). This led to a total sample size of eleven chimpanzees (n=4 at Chimfunshi Wildlife Orphanage; n=7 at Kristiansand zoo). Each chimpanzee included in the study (except the mother-infant pair) was individually tested in order to ensure that if any chimpanzee performed the target behavior(s) (flake manufacture or use), this would not render the other chimpanzees nearby unsuitable for further testing (given that potential observers could not be considered task-naïve anymore). Thus, our experimental design allowed us to confidently conclude that any occurrence of the target behaviors during testing must have been individually learned and not copied from (or elicited by) others. Furthermore, we tested chimpanzees in two populations in order to a) self-replicate our findings; b) slightly vary specifics of our methods in order to maximize the chances of occurrence of the target behaviors; and c) account for potential inter-group differences in housing and rearing conditions.

Subjects were housed at Chimfunshi Wildlife Orphanage and Kristiansand Zoo. Chimfunshi Wildlife Orphanage is located in Zambia, in the Copperbelt region (12° 23’ S, 29° 32’ E). Four chimpanzees (mean_age_ =29.5, range_age_: 18–46, 2F & 2M; Table S1 in Extended Data) were tested individually at Chimfunshi between 9:00 and 16:00 in July 2016. During tests, one chimpanzee was called into the management area while the other chimpanzees remained in the outdoor enclosure. The chimpanzees had access to a small caged outdoor enclosure (approx. 75 m^2^) and four indoor management areas. The outdoor enclosure was a fenced area with natural soil, vegetation and a few small pebble-like stones. The chimpanzees spent the day primarily in the outdoor enclosure, and slept in the inside management rooms. The chimpanzees were regularly provided with several enrichment devices such as cardboard tubes with food inside that they could retrieve using small twigs or their hands and teeth. Two daily feeds were provided between 11:30 and 12:30 and between 14:30 and 16:30. Three of the chimpanzees in this group (Milla, Cleo and Chiffon) spent some of their lives in close contact with humans. The specific details of their experiences with humans are currently unknown, but all three chimpanzees were retrieved from human owners, after having lived with them for at least a couple of years. Milla, for example, was rescued from a bar where she was kept as amusement for the patrons. Therefore, it is possible that some, or all, of these three subjects were enculturated to a certain (unknown) degree before they arrived at Chimfunshi. It is also possible that whilst they lived with humans, these three chimpanzees were kept in ‘deprived’ conditions (see also Henrich & Tennie, 2017). Therefore, the enculturation or deprivation status of these three subjects cannot be confidently assessed. The living conditions experienced by the chimpanzees before they were brought to the sanctuary might have affected their performance in our study. However, Colin (the fourth chimpanzee in the group) is Cleo’s son, and was born and raised at Chimfunshi, meaning that he can be considered unenculturated and un-deprived.

Kristiansand Zoo is located in Kristiansand, Norway. Seven mother-reared, unenculturated chimpanzees (mean_age_=23.7, range_age_: 7–41, 4F & 3M; Table S1 in Extended Data) were tested individually at Kristiansand Zoo (except a female, Jane, and her dependent offspring, which were tested together) in their sleeping quarters during the morning cleaning routines between 7:30 and 8:30 in May, June and November 2018. During this time, each individual was kept in a sleeping quarter, separated from the other chimpanzees by brick walls that prevented visual contact between the chimpanzees. Outside of cleaning hours, the chimpanzees at Kristiansand Zoo had access to two enclosures (one indoors and one outdoors). The indoor enclosure was equipped with several enrichment devices commonly found in zoological institutions such as an artificial termite mount regularly baited with honey, climbing frames, an automated puzzle feeder that periodically released nuts into a maze which the chimpanzees could obtain using tools, and a hanging log with holes baited with food rewards. The outdoor enclosure was an island of 1840 m^2^ surrounded by a water-filled moat, with natural soil and vegetation. The outdoor enclosure did not include any stones as the keepers removed them to prevent the chimpanzees from throwing them at the visitors. The indoor sleeping area was off-exhibit. Two daily feeds were provided at 10:00 and 14:00. Food was also scattered at 9:30 in the indoor and outdoor enclosures. It was decided *a priori* that only unenclturated chimpanzees that participated in all the test trials would be included in the study. All individuals entered the testing rooms voluntarily, and therefore could choose not to participate in the experiments.

**Figure 1:**
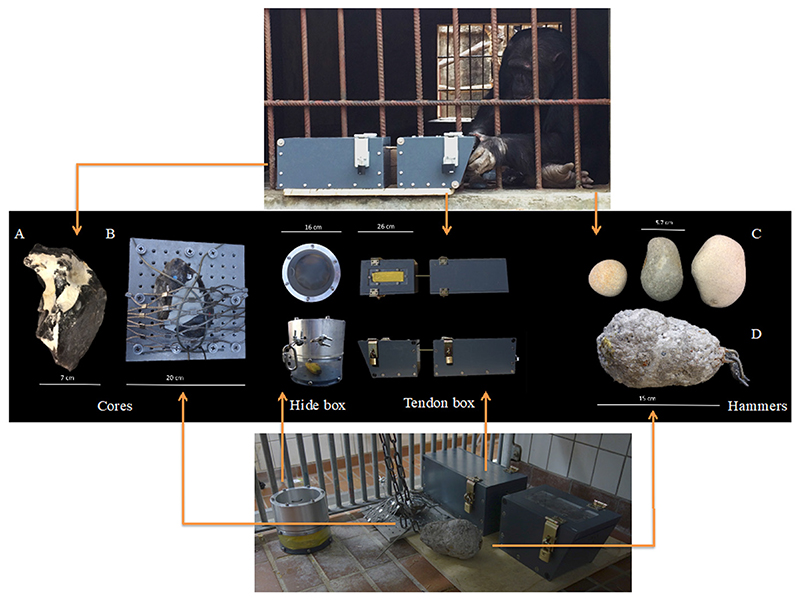
Experimental set-up. Testing materials used in Chimfunshi Wildlife Orphanage (core A, tendon box and hammers C) and Kristiansand Zoo (core B secured to the mesh of the enclosure, hide box, tendon box and hammer D). At Chimfunshi (top picture), individuals were provided with a loose core (**A**), the baited “tendon box” (where a rope acted as a tendon substitute) and three loose hammerstones (**C**). At Kristiansand Zoo (bottom picture), individuals were provided with a fixed core (**B**), the baited “hide box”, the baited tendon box and an artificial hammer (**D**). Both boxes were modeled after those used in the previous ape flaking studies and the food rewards contained within could only be obtained using a cutting tool. The arrows link each chimpanzee population with the materials provided during the experiments (middle panel).

Two different puzzle boxes baited with food items were used in order to motivate the chimpanzees to make (and subsequently use) flakes. Both puzzle boxes used in this study were novel for all the tested chimpanzees. However, the chimpanzees at both institutions were familiar with test apparatuses in general. Indeed the provision of the puzzle boxes served as part of the enrichment routine practiced in the testing facilities. In both testing institutions we used a puzzle box with a rope lock inspired by previous flaking experiments with great apes (e.g., Schick et al., 1999; Toth et al., 1993; Wright, 1972) that we named the “tendon box” (Figure 1). The tendon box was used to simulate a scenario in which, faced with an animal carcass, a subject must cut through taut tendons (a rope in our experiment) in order to dismember a body. Our tendon box consisted of two opaque boxes secured to a wooden board [box one (rewarded box): 26 × 17.3 × 17.3 cm; box two (non-rewarded): 36 × 15 × 17.2 cm]. The tendon box had a clear Plexiglas window (5 × 16 cm) at the top that allowed the reward inside to be visible to the chimpanzees. The door of the box was pulled shut by a rope that ran through the inside and exited through a hole in the opposite end. The rope then ran between the two boxes for approximately 5 cm and entered the second, non-rewarded box. The rope was secured in the non-rewarded box to a clamp that could be tightened to ensure that the rope was taut. The rope was only accessible in the area between the two boxes, and had to be cut there in order to open the door of box one. The rope was a brown twisted cord hemp rope, approx. 2 mm thick. This type of rope was selected as it was found to be (after pilot testing by EB) strong enough to withstand attempts by a human at opening the box without a tool but could be cut using a knife or flake. Collectively, the box weighed approximately 21 kg (including the board on which the boxes were fixed).

We also used a second puzzle box in Kristiansand Zoo named the “hide box”. The hide box design was inspired by the additional box used in the bonobo knapping experiments of Toth et al. (1993) as well as in the capuchin monkeys knapping experiments of Westergaard & Suomi (1994). This box (which was developed after data collection at Chimfunshi) roughly resembled a drum with an occluding silicone membrane 2 mm thick on top (Figure 1) and a transparent Plexiglass cylinder (16 cm wide × 15.5 cm high) with a metallic rim. The silicone membrane was screwed in between the cylinder and the rim, blocking the access to the reward placed inside the cylinder. The hide box was then secured to the bars of the rooms where the experiments took place (Figure 1).

The use of puzzle boxes baited with food is a common practice in cognitive experiments investigating animal’s problem solving abilities (e.g., Buttelmann et al., 2013; Forss et al., 2020; Hopper et al., 2007; Hopper et al., 2008; Whiten et al., 2005; Whiten et al., 2007). The rewards (baits) placed inside the two puzzle boxes included in our experiments consisted of peanuts and animal biscuits in Chimfunshi and half a banana or a yogurt in Kristiansand. The rewards were chosen based on the advice provided by the keepers regarding which were the preferred foods of the chimpanzees at each site.

In Chimfunshi, the chimpanzees were provided with three oval loose hammerstones (small, medium and large) in each trial (weight range 0.5–1 kg). Hammerstones were collected from streams around Birmingham, UK, based on the size and shape (similar to a potato) of the ones most commonly found in archaeological assemblages (Mora & De la Torre, 2005). Due to safety regulations, it was not possible to provide loose hammerstones to the chimpanzees housed at Kristiansand Zoo. Instead, one concrete rounded hammer (ca. 15 cm long × 10 cm wide, weight 2.2 kg) was provided during each trial. The weight of the hammer was modeled based on the hammers used by wild chimpanzees to crack nuts (Biro et al., 2003). The hammer was built around a metallic scaffold linked to a chain that allowed us to fix it to the bars of the testing rooms so the chimpanzees could not carry the hammer into the indoor enclosure. The concrete used to make the hammer included particles of up to 1 cm in diameter (Figure 1). The hammer was covered with non-toxic transparent epoxy resin to prevent its surface from disintegrating upon hammering.

In both sites, retouched Norfolk Chert cores were provided to the chimpanzees alongside the hammers (Figure 1). Unworked cores were purchased from a provider (Needham Chalks) in the UK and then knapped at the University of Birmingham. The cores were partially decortified to make the actual flint accessible, and in order to create platform angle variability between ~90 degrees and ~30–40 degrees which would make flake removal possible at the outset and without the need of manipulating the hammers by means of precision grips (similar to the procedure used in the earlier knapping studies; Toth et al., 1993; Westergaard & Suomi, 1994; Wright, 1972). This preparatory step was undertaken to account for the fact that chimpanzee hand morphology allows them to engage in power grips but prevents them from using forceful precision grips (Rolian & Carvalho, 2017). During the decortification process we aimed to produce either i) three separate surfaces with varying angles from which flakes could potentially be struck or ii) a continuous edge around the perimeter of the core with continuously varying angles within the abovementioned platform angle range. The cores weighed between 0.8 and 1.5 kg. Subjects received one core per trial and if the core was not modified, the core was used in further trials. In Chimfunshi, cores were provided loose to the chimpanzees and therefore could have been reduced with various techniques. Due to safety regulations, in Kristiansand Zoo the core had to be fixed on a metallic platform (20 × 20 × 2 cm) to prevent the chimpanzees from carrying the core into the indoor enclosure. Similarly to the previous orangutan experiment (Wright, 1972), the stationary position of the core limited the possible techniques of flake removal. The core was attached to the metallic platform using a metallic wired mesh with openings 5 cm wide and 3 mm wire (XTEND, Carl Stahl ARC GmbH, Architectural Cables and Mesh Systems), leaving a knappable section of the core (with a platform angle of less than 90 degrees) exposed.

In Chimfunshi, the tendon box was placed on a ledge outside of the testing area and baited before the subjects entered the room. This set up was chosen to increase the visibility of the tendon box from the experimenter’s location 3 m away from the room’s bars. Three hammerstones and one core were placed – all unfixed – on the floor inside the enclosure, allowing the chimpanzees to freely manipulate them. One camera (Sony HDR-CX330E Handycam) was set-up one meter from the enclosure, and recordings started once a subject entered the testing area. In Kristiansand, all testing materials (hide and tendon boxes, the artificial hammer and the fixed core) were placed inside the testing room and secured to the bars of the enclosure. All materials had to be placed inside the testing room because the chimpanzees could not extend their arms through the bars (as in Chimfunshi) due to safety reasons. Two Sony HDR-CX330E Handycams were set-up half a meter from these bars, and started recording once the subject entered the testing room. Potential tools were cleared from the testing areas before the tests started. However, the chimpanzees often brought tools with them into the testing quarters at the start of the tests.

### Experimental design

We implemented two experimental conditions: a baseline and a flake condition. During the baseline the subjects were provided with the testing materials but no demonstrations, guidance, or artefacts (e.g., no pre-made flakes) were provided. Crucially, no information regarding how to manufacture or use flakes was given before or during this condition. The aim of the baseline condition was to investigate whether chimpanzees could individually learn flake manufacture and use (as they were required to make a flake before they could use it). In Kristiansand Zoo, the baseline condition was split into two other sub-conditions (baseline condition I and baseline condition II) to control for the potential effect of testing with two baited boxes instead of one (as in Chimfunshi). During the baseline condition I, seven chimpanzees were provided with the tendon box, the hide box, a hammer and a fixed core (Figure 1). All chimpanzees in Kristiansand Zoo were tested individually in three trials each during the baseline condition I (condition duration range 01:05:40 to 03:00:49). We included a second baseline in Kristiansand Zoo to focus the attention of the individuals on solving a single task by only providing them with one box. In the baseline condition II, only the four most engaged individuals (two males and two females) of the seven that participated in the baseline condition I were tested. The box that each individual was tested with (tendon or hide box) was assigned randomly in baseline condition II. These four individuals were tested in three additional trials each during the baseline condition II (condition duration range 01:18:50 to 03:31:12). The individual trial length varied (range 00:29:14 to 02:02:19) depending on the duration of the local cleaning routines, as this was the time when testing took place. The same four individuals that we tested in the baseline condition II were further tested in the flake condition (condition duration range 01:46:23 to 02:38:32).

In the flake condition we used the same materials as in the baseline condition but we also provided the chimpanzees with a pre-made flake. The aim of the flake condition was to test if the chimpanzees could spontaneously recognize a flake as a potential cutting tool to access the puzzle boxes. The flake provided during this condition was made out of chimpanzees’ sight by the experimenters using freehand percussion. In Chimfunshi the flake measures were: platform depth = 8.46 mm, platform width = 21.46 mm, technological length = 50.76 mm and flake width = 47.56 mm. In Kristiansand the flake measures were: platform depth = 10.93 mm, platform width = 25.9 mm, technological length = 61.73 mm and flake width = 42.36 mm. Platform depth was measured as the distance from the impact point along the platform surface to the exterior margin of the flake and perpendicular to the interior surface of the flake. Platform width was measured from one lateral margin of the platform to the other. Flake length was measured from the impact point to the most distal point of the flake and flake width as the distance between the two flake edges at the midpoint and perpendicular to the length axis. Before the start of the flake condition, the experimenters tested the functionality of the flakes by opening the puzzle boxes themselves (only flakes that could cut open the puzzle box were provided). Each flake was placed unfixed (loosely on the floor) next to the hammerstone(s), core and puzzle box(es) before the subjects were allowed into the testing rooms.

All four chimpanzees at Chimfunshi were tested in the baseline condition II setup (with the tendon box) and the flake condition. Each trial lasted 20 minutes. The four chimpanzees at Chimfunshi were tested in three trials of the baseline condition II (60 min in total per individual) and in two trials of the flake condition (40 min in total per individual).

### Coding

From each video-recorded trial we coded i) the duration of the interactions (time spent in physical contact with the testing materials, from when the subject started contact until it paused for more than 3s or changed activity), ii) which testing material the chimpanzees interacted with and iii) if the interaction was manual or using a tool (objects other than the provided stones are subsumed under the ‘tool’ category, including objects that the chimpanzees brought into the enclosures themselves).

### Flake data capture

The two flakes provided during the flake condition were scanned with an Artec Space Spider 3D scanner using the data capture software Artec Studio 14 (Figure S1 in Extended Data). Similar scans could be created using photogrammetric approaches with freely available software like VisualSFM (Wu, 2011).

### Statistical analysis

A proportion (20%) of the interactions between the chimpanzees and the testing materials across experimental conditions from each institution were re-analysed by a second coder naïve to the goals of the experiment in order to assess the inter-rater reliability (one coder was used for each testing institution, so two separate second-coders recoded 20% of each data set, respectively). The second coders (AC & LK) were asked to re-code the videos based on a provided ethogram (Table S2 in Extended Data). The clips of the interactions provided to the second coders were randomly selected using a number generator and a number of dummy clips, where no interaction took place (10% of the total number of interactions), were included as a control. The second coders’ data was compared to the original coding using Cohen’s Kappa statistic. No statistical comparisons between individuals of the two housing facilities or experimental conditions were conducted.

To determine whether our sample size was suitably powered to test for the ability to manufacture and use flakes, the probability of the chimpanzees in our study *not* performing the target behaviors was calculated from a binomial probability distribution using the function dbinom from the R software version 3.6.1 (2019-07-05). The expected probability of the behaviors in the population was obtained from the only previous study that tested the spontaneous flake making and using abilities of naive, unenculturated primates (Westergaard & Suomi, 1994). Westergaard & Suomi (1994) found that 54% of the tested subjects (6/11) spontaneously detached flakes from a provided core whereas 20% of the tested subjects (3/15) used stones to cut open the provided puzzle boxes. For our power analysis (see Figure S2 in Extended Data) we used the incidence of flake making and use in naïve, unenculturated capuchin monkeys (Westergaard & Suomi, 1994) as the probability that naïve, unenculturated chimpanzees would also innovate these behaviors. If we had based our analysis instead on the previous incidence of flake making and using of enculturated and trained apes, the expected probability of the behavior would have been 100% (based on the results of Toth et al., 1993; Wright, 1972).

### Ethics

The experiments reported comply with the Guide for the Care and Use of Laboratory Animals (National Research Council, 2011), the American Society of Primatologists’ Principles for the Ethical Treatment of Primates, and with current Norwegian laws. The experiments were approved by the Ethical commission of the European Research Council (ERC). This study was further approved by the ethical board of Kristiansand Zoo before its commencement. The research at Chimfunshi was approved by the University of Birmingham research board (reference UOB 31213), in line with the requirements for testing of animals in the UK and internationally. The project was also approved by CRAB (Chimfunshi Research Advisory Board). All participation in the study was voluntary. The subjects were called by name into the testing quarters and could choose not to enter the testing rooms, and thus not participate in the study. If the subject chose to enter the testing quarters and thus participate in the study, the chimpanzees were free to interact as much or as little as they wanted with the testing materials. The experimenters never attempted to encourage the chimpanzees to interact or manipulate the testing materials, but merely observed their behavior during the trials. If the subjects showed any signs of distress or tried to exit the testing room by manipulating the door during testing, they would immediately be released back into their main enclosure. Subjects were never food or water-deprived, and continued with their regular feeding routine during the study. Subjects had access to water *ad libitum* prior, during, and after testing. The chimpanzees included in the study were used to being separated from their group for short periods of time during cleaning routines or veterinary check-ups. Therefore, the keepers and the research boards of both testing institutions agreed that the separation of the chimpanzees for this study would not cause any harm or distress to the chimpanzees. Nevertheless, the experimenters were always present during the experiments (alongside one chimpanzee keeper) and would have terminated the trial immediately if the chimpanzees had shown any signs of distress (this never occurred during the present study). No incidents or adverse events occurred during data collection for the present study.

## Results

There was a substantial to almost perfect agreement (Cohen, 1988) between the two coders of the interactions between the chimpanzees and the testing materials at Chimfunshi (k=0.684) and Kristiansand Zoo (k=0.947; Motes-Rodrigo & Bandini, 2021). Regarding the chimpanzee’s spontaneous knapping abilities, none of the chimpanzees included in our sample made flakes in either the baselines (when no information or final products were provided) nor in the flake condition (when a human-made flake was provided). In addition, no chimpanzee used the provided flake during the flake condition to open any of the baited boxes.

Modeled on the results from the only previous study that tested the spontaneous flake making abilities of naïve, unenculturated primates (Westergaard & Suomi, 1994), we found that the probability that we would not find flake making even once in our ape sample was 0.0002 whereas the probability that we would not find flake use in our sample was 0.085.

Although the chimpanzees in our sample did not make or use flakes, they interacted frequently with the testing materials by trying to open the puzzle boxes both by hand and using their teeth, thus proving motivated to retrieve the food rewards from inside the puzzle boxes (Table S3 in Extended Data). In Chimfunshi, the total, cumulative interaction time with the testing materials was 00:54:37, while at Kristiansand Zoo it was 02:05:21. These differences between sites are likely due to the longer trials and the larger number of individuals tested at Kristiansand Zoo compared to Chimfunshi. In Chimfunshi, the chimpanzees interacted the most with the tendon box (total interaction time 00:50:34) and the least with the flake (in the flake condition; total interaction time 00:00:25). In Kristiansand Zoo the chimpanzees interacted the most (total interaction time 01:35:18) with the hide box (the hide box was not available at Chimfunshi) and the least with the flake (in the flake condition; total interaction time 00:00:08). In Kristiansand Zoo, the chimpanzees interacted with the tendon box for a total of 00:12:53. Interactions were made both by hand and using tools (Table S4 in Extended Data). The chimpanzees used straws, plastic hose fragments, plastic cups, sticks and plastic pieces that they retrieved on their own as tools to try to open the puzzle boxes. However, the chimpanzees were never successful in opening the boxes. Instead, at both testing institutions the chimpanzees used these materials to try to lever open the lid of the tendon box and to probe different part of the boxes. Chimpanzees at both institutions knocked (touched repeatedly and in quick succession an object with the knuckles), slapped (touched in a fast movement an object with the palm of the hand) and hit (touched fast and using considerable force an object with any part of hand other than the palm) the testing materials provided. However, no percussive actions with a tool (e.g. hammers) took place in any of the trials.

## Discussion

In contrast to the earlier ape flaking studies using tutored, enculturated apes, none of the chimpanzees we tested made or used a flake during the baseline conditions or after being given a pre-made functional flake (flake condition). The same negative findings were obtained from the eight unenculturated, mother-reared chimpanzees as from the three chimpanzees with uncertain degrees of enculturation included in our sample. We did not observe sharp-edged stone tool making or use despite the fact that the chimpanzees seemed motivated to open the puzzle boxes, as suggested by their attempts to open the boxes both with and without tools. The use of tools other than a flake to try to open the puzzle boxes suggests that the chimpanzees did not perceive the flake as a potential tool. It is possible that the novelty of stone as a material compared to the familiar plastic and stick tools prevented the chimpanzees from innovating the use of stone as a tool during the experimental period (Gruber, 2016). It is unlikely that the notable absence of flake production and use in our study as compared to previous ape flaking studies is due to inter-species differences in cognitive and/or physical abilities. Cognitively, chimpanzees are at least on par in physical skills with orangutans and bonobos as they show by far the most extensive tool-use repertoires of all wild apes (which includes lithic percussive behaviours; Whiten et al., 1999; Whiten et al., 2001). Chimpanzees are also physically able to produce flakes, as evidenced by several reports of wild chimpanzees unintentionally detaching flake-like objects while engaging in nut-cracking using stone hammers and anvils (Carvalho et al., 2008; Mercader et al., 2007; Mercader et al., 2002). Although their hand morphology would have prevented the chimpanzees in our study from making sharp-edged stones using forceful precision grips in wich the thumb is opposed to the other fingers (Rolian & Carvalho, 2017), the tested chimpanzees could have employed power grips similar to those described in the context of other chimpanzee stone behaviors such as nut-cracking (Boesch-Achermann & Boesch, 1993). Therefore, we do not believe that hand morphology prevented the chimpanzees from enganging in percussive behaviors that might have led to sharp-stone making.

A more likely explanation for the discrepancy between the results of our study and those of previous ape flaking studies is the background and experiences of the subjects, both in the long term and immediately before testing took place. Contrary to the apes tested in the early flaking experiments, most of the chimpanzees included in our study (at least 8 out of 11 tested) were not enculturated nor had been exposed to human training in this or related tasks. Furthermore, all chimpanzees in our study were untutored in the target behaviours as they were not provided with social demonstrations of how to make or use flakes, whereas all previously tested apes (Toth et al., 1993; Wright, 1972) were exposed to human demonstrations (and sometimes even molding) before testing. It is therefore likely that enculturation had a large direct or indirect (via demonstrations) role in driving the findings of earlier reports of ape flake making and use. Not only do enculturated apes generally show skills and cognitive abilities different from unenculturated apes (e.g., Tomasello & Call, 1997), but previous studies have found that enculturation and/or extensive human training in certain tasks (e.g. Pope et al., 2018) lead apes to attend to and even in some cases copy demonstrated behavioural forms (Buttelmann et al., 2007; Tomasello et al., 1993). That is, the degree of human enculturation of the previously tested apes could have predisposed them to attend and perhaps reproduce the human demonstrations of flake making and use provided in these earlier studies (Toth et al., 1993; Wright, 1972). Alternatively, or in addition, enculturation could have increased the apes’ individual innovative skills to a degree that helped or allowed for the production and use of sharp-edged stone tools, prior to – and perhaps entirely independent from – demonstrations. With regards to unenculturated apes (including the majority of the chimpanzees tested in our study), these are unlikely to make and use flakes following human demonstrations given that the behaviours are not already expressed in baseline conditions (as unenculturated apes do not seem to copy novel behaviours beyond baseline performance; Clay & Tennie, 2018; Tennie et al., 2012; Tennie et al., 2020a; Tennie et al., 2020b).

Our results suggest that, outside the sphere of human enculturation (in combination with human demonstration and/or molding), the individual abilities of chimpanzees (and by phylogenetic proxy, that of hominins with chimpanzee-like cognitive abilities), do not seem sufficient to manufacture or use flakes. Assuming this interpretation is correct, there exist several possible evolutionary scenarios for the development of flake manufacture and use abilities in the hominid lineage. The first possible scenario is that hominin species pre-dating both the last common ancestor of chimpanzees and humans, as well as hominins with chimpanzee-like cognitive abilities, were able to intentionally manufacture and use flakes, but this ability was subsequently lost in the *Pan* lineage, and maintained in the hominin lineage. If this scenario did indeed occur, we would expect that hominin species that evolved after the hominin split from *Pan* (approximately seven million years ago) would have engaged in flake manufacture and use. However, there is a distinct absence of flaked stone tools in the archaeological record for millions of years after the split between hominins and the genus *Pan*. This gap in the archaeological record could be due to a very low density of manufactured flakes in the environment, which in addition to their archaic characteristics, would render their identification in archaeological excavations difficult (Semaw et al., 1997). Low flake manufacture densities during this period could be explained by a lack of necessity for flakes in the specific ecological niches inhabited by the different hominin species. An alternative scenario would be one in which hominoids with equivalent cognitive abilities to chimpanzees did not have the ability to make flakes. According to this scenario, the ability to manufacture flakes would have evolved later in the hominin lineage (and then may or may not have remained dormant), resulting in certain hominin species eventually crossing the cognitive Rubicon for flake manufacture and use. In both of these scenarios, it remains an open question whether learning mechanisms involved in modern human cultural transmission (especially copying social learning mechanisms; Tennie et al., 2017) were responsible for the acquisition of flake manufacture and/or use abilities.

Flake manufacture and use independent of copying social learning has already been suggested to be the most likely explanation for a phylogenetically independent case of flake manufacture and use in task-naïve, unenculturated (G. Westergaard, pers. comm) capuchin monkeys (Westergaard & Suomi, 1994). Two previous studies tested the spontaneous flaking abilities of capuchins monkeys (*Sapajus apella*; Westergaard & Suomi, 1994; Westergaard & Suomi, 1995). To examine the spontaneous flaking abilities of this species, Westergaard and Suomi implemented a similar methodology to our baseline condition by providing task-naïve, unenculturated capuchins with the necessary materials (hammers and cores) and motivation (puzzle box baited with food similar to the hide box) to make and use flakes (Westergaard & Suomi, 1994). In contrast to the chimpanzees tested in our study, some capuchins spontaneously made (6/11) and used flakes (3/15) in baseline conditions, thus validating the experimental paradigm used in our study. As the tested capuchins were naïve to flake manufacture and use prior to and during testing (i.e. no demonstrations were provided) this capuchin study represents a proof of principle that the abilities to manufacture and use flakes as cutting tools in at least some species of tool-using primates can develop in the absence of copying opportunities and enculturation (Westergaard & Suomi, 1994).

The present study strongly suggests that contrary to the capuchins, naïve untrained chimpanzees do not possess or develop flake making and using abilities spontaneously. Such inter-species differences could perhaps be explained by different genetic predispositions for stone manipulation in capuchins and chimpanzees (Hayashi, 2015). For example, previous studies have shown that capuchins spontaneously manipulate novel objects by hitting them against hard substrates (Brunon et al., 2014). Taken together, the results of the present study, the capuchin data and the archaeological record, support a scenario in which the abilities to make and use flakes would have evolved independently, and at least twice, during primate evolution: in capuchins (see also Proffitt et al., 2016 for flake production in wild capuchins) and at least once in the hominin lineage once cognitive abilities more advanced than those found in chimpanzees developed and/or the ecological pressure for sharp tools emerged. This scenario would also explain the large time gap (spanning several million years) between the split of hominins and *Pan* and when the first flaked stone tools appear in the archaeological record (currently circa 2.58 million years: Braun et al., 2019).

## Supplementary Material

Full Text XML

Supplementary Material

Extended Data

## Data Availability

Open Science Framework: Naïve, unenculturated chimpanzees fail to make and use flaked stone tools. https://doi.org/10.17605/OSF.IO/5UWKA (Motes-Rodrigo & Bandini, 2021). This project contains the following underlying data: - coding stonecult AMR kristiansand.csv (Data coded from the experiments conducted at Kristiansand Zoo) - coding stonecult EB chimfunshi.csv (Data coded from the experiments conducted at Chimfunshi Wildlife Orphanage) - second_coder_data_louise.csv (Data coded by the second coder from Kristiansand Zoo) - second_coder_data_MB.xlsx (Data coded by the second coder from Chimfunshi Wildlife Orphanage) Open Science Framework: Naïve, unenculturated chimpanzees fail to make and use flaked stone tools. https://doi.org/10.17605/OSF.IO/5UWKA (Motes-Rodrigo & Bandini, 2021). This project contains the following extended data: - Dictionary variable names.docx - Extended data figures and tables.docx - Power simulations Bandini, Motes-Rodrigo *et al.* R - stonecult_SubKnerten_ConFlaketendon_s23_t44_Cam2. mp4 - stonecult_SubJosefine_ConFlakedrum_s22_t42_Cam1. mov - Chimfunshi_SubChiffon_ConBaseline.MPEG Data are available under the terms of the Creative Commons Attribution 4.0 International license (CC-BY 4.0).

## Revised: Amendments from Version 1

In this revised version of our manuscript we have addressed the comments provided by the three reviewers as well as made some changes throughout the manuscript to improve clarity and readability. These changes mainly involve clarifications of concepts and provision of additional methodological information. For example, we have now clarified which mechanisms are encompassed by the term copying social learning and what behaviours were subsumed by the “tool” category during video coding. We have also specified that precision grips were not required to solve the task we implemented and we have provided further and more detailed information on the rearing background and the previous experience with stones of the chimpanzees included in the study. In this version of the manuscript we have conducted two different power analyses for the two behaviours tested (stone tool making and stone tool use) to replace the previous power analysis which considered simultaneously stone tool making and stone tool use. We have rewritten the first part of the discussion to include another possible explanation for our negative findings in stone tool use, namely that chimpanzees did not use stones because this was a novel material which wasn’t preferred over more familiar materials such as sticks or plastic. Finally, we have also discussed how the differences in hand morphology between stone-tool making and using hominins and chimpanzees influence the interpretation of our results.

**Any further responses from the reviewers can be found at the end of the article**

## Plain language summary

One of the first types of tools found in the archaeological record are sharp-edged stones. How tool-using hominins learnt to make and use these tools is still debated. One way to study how hominins might have learnt to make and use stone tools is to test our closest living relatives, chimpanzees. In this study, we aimed to elicit stone tool making and use by providing 11 untrained chimpanzees (most of whom were mother-reared) housed at two different institutions with baited puzzle boxes that could only be opened with a sharp tool. The chimpanzees were also provided with stone cores and hammerstones that they could use to make the sharp stone tools necessary to open the puzzle boxes. No demonstrations on how to make or use stone tools were provided to the chimpanzees before or during testing. Although the chimpanzees were motivated and interested in accessing the puzzle boxes, none of the subjects in this study spontaneously made or used sharp stones. Our findings suggest that without extensive human training and/or demonstrations, chimpanzees cannot learn how to make or use sharp stone tools by themselves. These results are consistent with the hypothesis that the ability to make and use sharp stone tools may have developed in our own lineage after the split from our last common ancestor with chimpanzees, approximately seven million years ago.
