# Supplementary material for: Naïve, unenculturated chimpanzees fail to make and use flaked stone tools [version 2; peer review: 3 approved]
Source: Open Res Eur. Author manuscript; Available in PMC 2022 Mar 3. (PMC7612464)
Supplement: Extended Data [file EMS129479-supplement-Extended_Data.zip › Dictionary variable names.docx]

Dictionary variable names

## Coding sheet: coding stonecult AMR kristiansand.csv

| variable name | description |
| --- | --- |
| cond_trial | individual trial number |
| individual | name of the individual chimpanzee |
| condition | name of the experimental condition |
| duration_bout | duration of the event |
| duration_trial | total duration of the trial |
| interact_w | testing materials that the chimpanzees interacted with in a particular event |
| subpart | subpart of the testing material the chimpanzees interacted with |
| how | type of manipulation of the testing materials |
| part | bodypart or tool type that was involved in the event |
| bodypart_tool | whether the interaction involved a tool or not |
| site | housing facility where the experiments took place |
| session.n | session code used to label the video recordings of the experiments |
| trial.n | trial code used to label the video recordings of the experiments |

## Coding sheet: coding stonecult EB chimfunshi.csv

| variable name | description |
| --- | --- |
| day | day in which the trial took place |
| month | month in which the trial took place |
| year | year in which the trial took place |
| cond_trial | individual trial number |
| individual | name of the individual chimpanzee |
| condition | name of the experimental condition |
| start_bout | time in video where the event stats |
| end_bout | time in video where the event ends |
| duration_bout | duration of the event |
| duration_trial | total duration of the trial |
| interact_w | testing materials that the chimpanzees interacted with in a particular event |
| Manip_type | type of manipulation with the testing materials |
| set_up | whether the individuals where tested individually or in group |

## Coding sheet: second_coder_data_louise.csv

| variable name | description |
| --- | --- |
| sequential_n | event ID used to select via random number generator the subset of events recoded by the second coder |
| individual | name of the individual chimpanzee |
| condition | name of the experimental condition |
| start_bout | time in video where the event stats |
| end_bout | time in video where the event ends |
| duration_bout | duration of the event |
| interact_w | testing materials that the chimpanzees interacted with in a particular event |
| subpart | subpart of the testing material the chimpanzees interacted with |
| part | bodypart or tool type that was involved in the event |
| session.n | session code used to label the video recordings of the experiments |
| trial.n | trial code used to label the video recordings of the experiments |
| duration_bout2 | duration of the manipulative event in seconds |
| interact_w2 | testing materials that the chimpanzees interacted with in a particular event, coded by the second coder |
| subpart2 | subpart of the testing material the chimpanzees interacted with, coded by the second coder |
| part2 | bodypart or tool type that was involved in the event, coded by the second coder |
|  |  |
