# Supplementary material for: Naïve, unenculturated chimpanzees fail to make and use flaked stone tools [version 2; peer review: 3 approved]
Source: Open Res Eur. Author manuscript; Available in PMC 2022 Mar 3. (PMC7612464)
Supplement: Extended Data [file EMS129479-supplement-Extended_Data.zip › Extended data figures and tables.docx]

Naïve, unenculturated chimpanzees fail to make and use flaked stone tools

**Supplementary Materials and Methods**


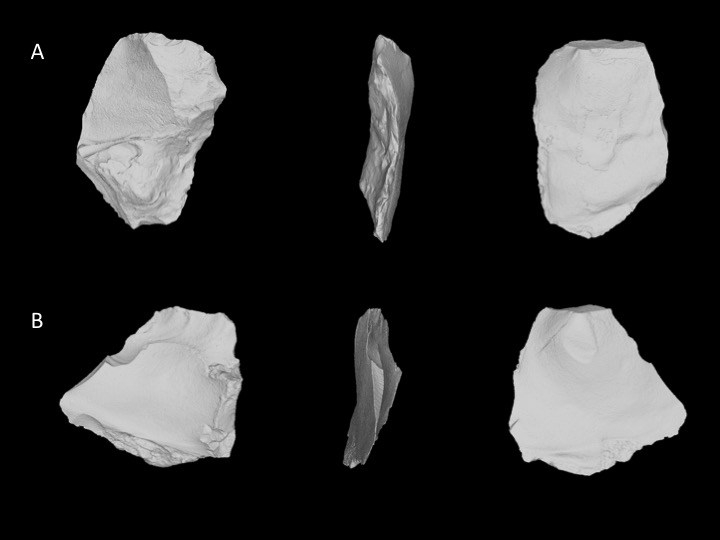


Figure S1: 3D models of the flakes provided during the Flake condition at Chimfunshi Wildlife Orphanage (A) and Kristiansand Zoo (B). Both flakes were made by the experimenter prior to testing and out of sight of the chimpanzees.


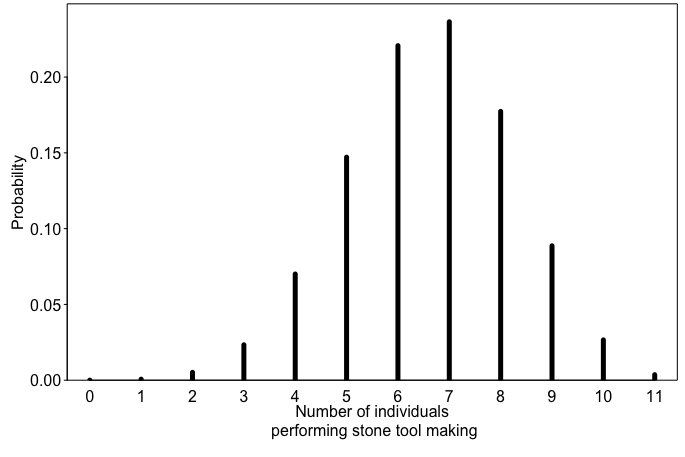


Figure S2. Calculated probabilities of finding different numbers of individuals showing stone tool making behavior in our pooled sample. We assumed that the expected incidence of the behavior in the population was 60% based on a previous flaking study conducted with unenculturated, task-naïve capuchin monkeys (Westergaard and Suomi 1994). Depicted probabilities were then drawn from a binomial probability distribution using the R function dbinom.

Table S1: Demographic data on the chimpanzees included in the study.

| Subject | Birth year | Sex | Site | Rearing conditions |
| --- | --- | --- | --- | --- |
| Dixie | 1977 | F | Kristiansand | Mother reared |
| Jane/Yr | 1999/2011 | F/F | Kristiansand | Mother reared |
| Josefine | 1983 | F | Kristiansand | Mother reared |
| Junior | 2003 | M | Kristiansand | Mother reared |
| Knerten | 2000 | M | Kristiansand | Mother reared |
| Tobias | 1994 | M | Kristiansand | Mother reared |
| Chiffon | 2000 | F | Chimfunshi | Mother reared |
| Cleo | 1983 | M | Chimfunshi | Hand reared |
| Colin | 1988 | M | Chimfunshi | Mother reared |
| Milla | 1972 | F | Chimfunshi | Hand reared |

Table S2: Ethogram of the behaviors coded from video recordings in each trial of each experimental condition.

| Behavior | Description |
| --- | --- |
| Touch the hide box | The chimpanzee touches the drum box with the hand or a tool |
| Touch the tendon box | The chimpanzee touches the rope box with the hand or a tool |
| Touch the hammer | The chimpanzee touches the hammerstone/concrete hammer with the hand or a tool |
| Touch the core | The chimpanzee touches the flint core with the hand or a tool |
| Touch the flake | The chimpanzee touches the flake with the hand or a tool |

Table S3: Frequencies and mean durations ± SD (between brackets) of the interactions with the different testing elements by individual chimpanzee across experimental conditions. Names in italics mark the chimpanzees from Chimfunshi. Superindexes indicate the individual’s sex.

|  | Tendon box | Core | Hide box | Flake | Hammer | Total |
| --- | --- | --- | --- | --- | --- | --- |
| Dixie^F^ | 0 | 1(1) | 115(7.90±9.76) | 0 | 7(3±2.89) | 123 |
| Jane^F^/Yr^F^ | 2(14±5.66) | 2(4.5±2.12) | 29(9.10±9.31) | 0 | 5(4.2±3.11) | 38 |
| Josefine^F^ | 40(10.3±10) | 2(38±50.9) | 48(18.4±26) | 1(3) | 25(14.8±17.1) | 116 |
| Junior^M^ | 12(9.9±6.5) | 1(1) | 29(6.7±7.4) | 0 | 1(1) | 43 |
| Knerten^M^ | 7(10.3±8.7) | 3(4.3±4.2) | 264(12.3±15.0) | 1(5) | 33(8.7±8) | 308 |
| Tobias^M^ | 21(6.9±5.8) | 2(3.5±3.5) | 35(6.5±5.9) | 0 | 24(9±8.5) | 82 |
| *Chiffon^F^* | 11(10.3±11) | 0 | 0 | 0 | 0 | 11 |
| *Cleo^M^* | 20(14.8±16.1) | 9(4±3.9) | 0 | 4(2.5±3) | 0 | 33 |
| *Colin^M^* | 51(42.3±179.9) | 17(6.4±6.8) | 0 | 0 | 0 | 68 |
| *Milla^F^* | 35(13.3±26.5) | 8(12.3±11.4) | 0 | 1(15) | 0 | 44 |

Table S4: Frequency and total time of the interactions with different testing materials using hands or tools across testing sites.

| Site | Testing material | Interact via | N | Total time  (sec) |
| --- | --- | --- | --- | --- |
| Chimfunshi | tendon box | hand | 110 | 2941 |
| Chimfunshi | tendon box | tool | 7 | 93 |
| Chimfunshi | core | hand | 17 | 117 |
| Chimfunshi | flake | hand | 5 | 25 |
| Kristiansand | tendon box | hand | 77 | 717 |
| Kristiansand | tendon box | tool | 5 | 56 |
| Kristiansand | core | hand | 11 | 107 |
| Kristiansand | hide box | hand | 515 | 5690 |
| Kristiansand | hide box | tool | 5 | 28 |
| Kristiansand | flake | hand | 2 | 8 |
| Kristiansand | hammer | hand | 95 | 915 |
