# Supplementary material for: Naïve, unenculturated chimpanzees fail to make and use flaked stone tools [version 2; peer review: 3 approved]
Source: Open Res Eur. Author manuscript; Available in PMC 2022 Mar 3. (PMC7612464)
Supplement: Supplementary Material [file EMS129479-supplement-Supplementary_Material.pdf]

Open Peer Review

Current Peer Review Status: ✔ ✔ ✔

Version 2

Reviewer Report 16 July 2021

https://doi.org/10.21956/openreseurope.15034.r27258

© **2021 Leca J.** This is an open access peer review report distributed under the terms of the Creative Commons Attribution License, which permits unrestricted use, distribution, and reproduction in any medium, provided the original work is properly cited.

✔

Jean-Baptiste Leca
[1] Department of Psychology, University of Lethbridge, Lethbridge, Canada
[2] School of Natural and Engineering Sciences, National Institute of Advanced Studies, Bangalore, India

I thank the authors for taking into account my comments and addressing my few and minor concerns. I am happy to state that the manuscript has significantly improved. I am now glad to recommend this manuscript for indexing.

Competing Interests: No competing interests were disclosed.

Reviewer Expertise: Since 2003, my main research program focuses on the mechanisms and evolution of material culture in non-human primates (more specifically macaques), with an emphasis on stone play and stone tool use.

I confirm that I have read this submission and believe that I have an appropriate level of expertise to confirm that it is of an acceptable scientific standard.

Version 1

Reviewer Report 18 May 2021

https://doi.org/10.21956/openreseurope.14256.r26750

© **2021 Graham K.** This is an open access peer review report distributed under the terms of the Creative Commons Attribution License, which permits unrestricted use, distribution, and reproduction in any medium, provided the original work is properly cited.

✔

Kirsty Graham
School of Psychology & Neuroscience, University of St Andrews, St Andrews, UK

This article is a much needed update to the question of whether nonhuman apes can make and use sharp stone tools. Previously we were left to decide whether we considered early studies of enculturated apes with incredibly small sample sizes (n=1 or 2) to be convincing evidence of this ability. Indeed, the authors take us through a step-by-step account of the training process involved in those “successful” experiments. They highlight the unusual conditions in which these apes were raised and how this may have contributed to their success. I have some specific comments about the manuscript below:

Introduction -
I wonder: had Kanzi been trained/taught to cut things before this experiment (e.g. using a knife)? He may have had a concept of “cutting” from past experiences.

I agree with the authors that it’s surprising that chimpanzees haven’t been tested before, given propensity for tool-use. A valuable addition to the literature!

Methods:
Early in the methods the authors introduce “a sanctuary and a zoo” before naming them further down. Either name them at the initial mention or state later on that Chimfunshi Wildlife Orphanage is the sanctuary as it may not be clear to all readers.

In the methods, it would be more helpful to give the n for the chimpanzees that were included, rather than an n for the total number of chimpanzees and then allow the reader to calculate how many the authors ended up with. Maybe change to something like “We aimed *a priori* to test all nine zoo-housed chimpanzees… However, once testing started, two zoo-housed chimpanzees were excluded from the study (n=7).” Otherwise at a cursory glance it seems that the sample size is inflated.

Either remove “Africa” after Zambia, or add “Europe” after Norway. Could also give a more specific location for Chimfunshi in Zambia. Otherwise, it’s a very good description of the conditions for the subjects in both locations.

Why was the “hide box” only used at the zoo and not the sanctuary?

Flake data capture is great! Very helpful. I also appreciated the thoroughness of descriptions for the coding and analysis for interobserver reliability, as well as the power analysis for the sample size. And the ethics! A lot to commend about how clear this section is.

Results and Discussion –
These sections were clearly written and easy to follow. The comparison to the capuchin study is very interesting, and presented in a way that is clearly relevant for the current study.

Is the work clearly and accurately presented and does it cite the current literature?
Yes

Is the study design appropriate and does the work have academic merit?

Yes

Are sufficient details of methods and analysis provided to allow replication by others?
Yes

If applicable, is the statistical analysis and its interpretation appropriate?
Yes

Are all the source data underlying the results available to ensure full reproducibility?
Yes

Are the conclusions drawn adequately supported by the results?
Yes

Competing Interests: No competing interests were disclosed.

Reviewer Expertise: Primatology, animal behaviour, gestural communication.

I confirm that I have read this submission and believe that I have an appropriate level of expertise to confirm that it is of an acceptable scientific standard.

Reviewer Report 17 May 2021

https://doi.org/10.21956/openreseurope.14256.r26751

© 2021 Leca J. This is an open access peer review report distributed under the terms of the Creative Commons Attribution License, which permits unrestricted use, distribution, and reproduction in any medium, provided the original work is properly cited.

Jean-Baptiste Leca
[1] Department of Psychology, University of Lethbridge, Lethbridge, Canada
[2] School of Natural and Engineering Sciences, National Institute of Advanced Studies, Bangalore, India

This is a well-written manuscript presenting an innovative, solid, and thorough experimental study on the ability (or lack thereof) to make and use flaked stone tools by captive, naïve, and unenculturated chimpanzees. The overarching goal of this research is to gain a better understanding of the cognitive processes underlying the emergence and evolution of lithic technologies in early hominins.

The topic was well introduced. The background literature was appropriately reviewed and critically addressed (but see below my few minor questions). The objectives were clearly laid out and ultimately reached – even though the results obtained by the authors were largely negative in the sense that the study subjects failed to express flake-making and flake-using behaviors. The methodological procedures were clearly described and extensively explained (but see below my few minor questions). The self-replication across two populations of chimpanzees was certainly a

bonus.

Overall, the layout is logical and the manuscript reads quite well. Findings from previous research were clearly summarized and put into perspective in light of this study (but see below my few minor questions). The authors did a good job at making a relatively complex study design simple, which makes the study accessible to a non-expert audience (while maintaining the interest of experts in this field!). I have no major issues with the outline/structure and conclusion of the manuscript. The (extended) figures, tables, and underlying data are informative and relevant. This is an interesting study that clearly fits an information gap, and has significant implications for the emergence of a major milestone in human evolutionary history.

Before I can recommend this manuscript for indexing, I just have a series of minor question/concerns (some of them are actually not central to the manuscript), which I anticipate the authors will be able to handle easily.

Page 1 (and Discussion section) I understand the phylogenetic relevance of the comparison between *Homo sapiens* and *Pan troglodytes* in specific behavioral domains to draw inferences on the evolution of specific cognitive abilities. In the context of stone tool-*making* (probably more than in the context of stone tool *use*), I am wondering to what extent some of your analyses should control for obvious inter-species differences in hand anatomy and their consequences in terms of manual dexterity, bimanual coordination, the magnitude of manual asymmetries, and variation in independent control over the digits. If it is not possible to analytically control for these variables, could you at least please elaborate on this confounder in your Discussion section?

Page 3: Could you please clarify the following statement about your study, by possibly making it more specific?
"Therefore, these findings suggest that without extensive human training and demonstrations, chimpanzees cannot learn how to make or use stone tools by themselves."
Are you suggesting that the stone-assisted nut-cracking behavior reported in several wild communities of chimpanzees across West Africa – where this activity has existed for at least 4,300 years (Mercader *et al*., 2007; Sirianni *et al*., 2015[ref-1]) – would not have been possible without any human influence?
Or did you mean "[...] chimpanzees cannot learn how to make or use *sharp-edged* stone tools by themselves."?

Page 3: Could you please specify what you mean by "some types of emulation"? What are the different forms of product-oriented copying, and among them, which ones "allow for the direct transmission of behavioural forms via the observation of [a model's...] products"?

Page 4 (or page 9): When explaining the severe limitations of research on encultured great apes, maybe you could cite a couple of relevant studies, such as Suddendorf & Whiten (2001: Psychol Bull)[ref-2]. In their paper, they stated that "Enculturation does have the potential to modify the cognitive abilities of apes" (p. 644). They found that these modifications concern the domains of both physical cognition (such as hidden displacement of objects but also tool use) and social cognition (such mind reading, including attributing intentions to others and imitation).
Alternatively, maybe you could elaborate on the study by Furlong *et al*. (2008) that you just cited. Even though their experiments are not about stone tool-making/use, their methodology systematically compared the performance of non-enculturated, semi-enculturated, and fully-

enculturated captive chimps in three raking tool tasks, and their results provided the first empirical evidence for the differential effects of enculturation on subsequent tool use capacities in captive chimpanzees.

Page 4: In light of your present results (about the failure to make and use flaked stone tools by naïve and unenculturated chimpanzees), how do you interpret positive findings of flake manufacture and use in untrained, unenculturated capuchin monkeys (Westergaard & Suomi, 1994) in an experimental paradigm that was similar to that used with chimpanzees? I understand your brief and speculative explanation in the Discussion section about “different genetic predispositions for stone manipulation in capuchins and chimpanzees” (page 10). However, considering the prevalence and diversity of stone-assisted percussive behaviors in wild and captive chimpanzees, I have to admit that I am not fully convinced by the interpretation that chimpanzees would be less prone to stone manipulation than capuchin monkeys. Could you please elaborate on this argument by using further empirical evidence from the literature?

Page 5: At both study sites, you stated that “rocks” were available in the outdoor enclosure. Could you please be more specific about the exposure to these obviously relevant lithic materials? Were they loose stones or embedded substrates? If the former, would you have an estimation of the density, average size, types of stones? Were the chimpanzees ever observed manipulating them? If so, what type of manipulation are we talking about here? (e.g., exploratory, playful, instrumental/tool use?)

Page 5: At both study sites, you stated that the chimpanzees were “familiar with test apparatuses in general” and were even provisioned with “puzzle boxes”. Could you please be more specific about the nature of these test apparatuses and puzzle boxes, other than the ones you provided to these study subjects?

Page 8: In the Methods, you first wrote “a second coder” and then, in the next sentence, you wrote “Two naïve coders”. Could you please clarify? Was there one second coder at one study site and another second coder at the other study site?

Is the work clearly and accurately presented and does it cite the current literature?
Yes

Is the study design appropriate and does the work have academic merit?
Yes

Are sufficient details of methods and analysis provided to allow replication by others?
Yes

If applicable, is the statistical analysis and its interpretation appropriate?
Yes

Are all the source data underlying the results available to ensure full reproducibility?
Yes

Are the conclusions drawn adequately supported by the results?

Yes

Competing Interests: No competing interests were disclosed.

Reviewer Expertise: Since 2003, my main research program focuses on the mechanisms and evolution of material culture in non-human primates (more specifically macaques), with an emphasis on stone play and stone tool use.

I confirm that I have read this submission and believe that I have an appropriate level of expertise to confirm that it is of an acceptable scientific standard.

Reviewer Report 15 April 2021

https://doi.org/10.21956/openreseurope.14256.r26632

© 2021 Moore M. This is an open access peer review report distributed under the terms of the Creative Commons Attribution License, which permits unrestricted use, distribution, and reproduction in any medium, provided the original work is properly cited.

Mark Moore
Archaeology and Palaeoanthropology, University of New England (UNE), Armidale, Australia

This is an important study into the ability of chimpanzees to spontaneously make and use stone flakes, and I recommend indexing with minor revisions.

The topic is important because some researchers see the emergence of stone-flaking as cognitively insignificant, while others see it as a sort of 'cognitive Rubicon'. An independent assessment of these competing models is possible through what the authors refer to as 'cognitive cladistics'—by studying the capabilities of the great apes, our closest living primate relatives. The authors assess prior experiments in great ape stone-flaking, and conclude that the results are of limited use for two reasons: 1) the tested apes were enculturated into human ways of learning because they were reared in a human cultural environment; and 2) stone-flaking and use was demonstrated to the tested apes. The behaviours observed in prior studies were therefore not 'spontaneous', and did not track 'naturally developing abilities', and are therefore of limited use in assessing the emergence of stone flaking. The authors' experiments in this paper were with unenculturated chimpanzees, and without prior demonstration, in order to test the hypothesis that flake manufacture and use were within the 'natural individual cognitive reach of apes'.

A premise of the present study is that the learning environment was key to the copying of stone flaking behaviour, as summarised in the paper's introduction. Therefore, the authors argue, the prior experiments were of limited value because the subjects were deliberately and extensively enculturated by their handlers. However, Eren *et al.* (2019,[1] not cited by the authors) suggest that it is 'incongruous' that the apes—particularly Kanzi, a bonobo—in these prior experiments were not able to mimic Oldowan stone tools given hominoid's technological propensities, suggesting instead that the apes were actually working in an impoverished learning environment. Eren *et al*. suggest that apes may indeed have the necessary cognition to make Oldowan stone tools, but that the 'misdirection' inherent in the demonstrations by human experimenters compromised the

ape's learning; and indeed, Eren *et al*. suggest that the demonstrations were 'minimal' (Eren *et al*. 2019:313), in contradiction to the present authors' interpretation of the same studies (although I note that the present authors express more ambivalence about the importance of 'demonstrations' in the discussion section than in the introduction). The authors should consider and respond to Eren *et al*.'s conclusion in developing the background to their experiments.

The experimental design followed aspects of prior work on this topic by using two types of baited boxes, and provisioning the rooms with one or more indentors (hammerstones) and a high-quality knapping stone with appropriate angles for flaking. In one set of experiments, the hammerstone was made of concrete and chained to the metal bars of the cage, and the core was secured in wire mesh. This effectively prevented a full and free manipulation of these objects, and would have stopped the sort of stone-fracturing events documented in Kanzi; that is, the chimpanzees in this set of the authors' experiments were not given full range to discover the affordances offered by the stones. However, the chimpanzees in the other set of experiments were given the stones unimpeded by chains and wire, yet those chimpanzees also did not make Kanzi-like stone-flaking manipulations. Because of this, although perhaps not ideal, I am happy with accepting this aspect of the experimental design. I note that all of the chimpanzees were given free manipulation of the provided flake in certain experimental iterations, yet did not discover the affordance offered by the sharp edge. The experimental design, controls, and data collection effectively support the authors' experimental conclusions that chimpanzees do not spontaneously flake stone to solve ecological problems.

One aspect of the discussion needs to be further elaborated, however. The authors state that the chimpanzees 'demonstrated an understanding of the requirements of the task' to remove the treats from the two types of containers, and were 'motivated to open the puzzle boxes'. Their evidence for this is the observation that the chimpanzees 'frequently interacted with' the lock/hide mechanisms. However, the authors' observations are vague and, as given, are insufficient for inferring 'understanding' and 'motivation'. More detail is necessary to support this point, as 'understanding' and 'motivation' are key in the epistemological chain that links the possible recognition of tool affordances to a goal. The ways that the chimpanzees used other objects in attempts to gain access to the boxes—referred to only briefly—may provide the necessary information if developed in more detail. How, specifically, were those objects used, and what does this say about the characteristics the chimpanzees thought those objects had, but were (apparently) not perceived in the stones? Perhaps there's a degree of object familiarity at play here.

Under 'coding', the authors should clarify that the tool-use referred to involved other objects the chimpanzees brought into the room, and not stone tools.

References

1. Eren MI, Lycett SJ, Tomonaga M: Underestimating Kanzi? Exploring Kanzi-Oldowan comparisons in light of recent human stone tool replication.*Evol Anthropol*. 2020; **29** (6): 310-316 PubMed Abstract | Publisher Full Text

Is the work clearly and accurately presented and does it cite the current literature?

Partly

Is the study design appropriate and does the work have academic merit?

Yes

Are sufficient details of methods and analysis provided to allow replication by others?
Yes

If applicable, is the statistical analysis and its interpretation appropriate?
Yes

Are all the source data underlying the results available to ensure full reproducibility?
Yes

Are the conclusions drawn adequately supported by the results?
Yes

Competing Interests: No competing interests were disclosed.

Reviewer Expertise: Stone tools, hominin cognitive evolution, the design space of stone flaking.

I confirm that I have read this submission and believe that I have an appropriate level of expertise to confirm that it is of an acceptable scientific standard.
