# Supplementary material for: Naïve, unenculturated chimpanzees fail to make and use flaked stone tools
Source: Open Res Eur. 2021 Jul 15;1:20. Originally published 2021 Mar 24. [Version 2] doi: 10.12688/openreseurope.13186.2 (PMC7612464; doi:10.12688/openreseurope.13186.2)
Supplement: Peer review history

# Peer review: Reviewer response for version 2

**DOI:** 10.21956/openreseurope.15034.r27259

**Published:** 2021-11-25

**Authors:** Kirsty Graham

**Affiliations:** 1 School of Psychology & Neuroscience, University of St Andrews, St Andrews, UK

I do not have any new comments.

Is the study design appropriate and does the work have academic merit?

Yes

Is the work clearly and accurately presented and does it cite the current literature?

Yes

If applicable, is the statistical analysis and its interpretation appropriate?

Yes

Are all the source data underlying the results available to ensure full reproducibility?

Yes

Are the conclusions drawn adequately supported by the results?

Yes

Are sufficient details of methods and analysis provided to allow replication by others?

Yes

Reviewer Expertise:

Primatology, animal behaviour, gestural communication.

I confirm that I have read this submission and believe that I have an appropriate level of expertise to confirm that it is of an acceptable scientific standard.

# Peer review: Reviewer response for version 2

**DOI:** 10.21956/openreseurope.15034.r27258

**Published:** 2021-07-16

**Authors:** Jean-Baptiste Leca

**Affiliations:** 1 School of Natural and Engineering Sciences, National Institute of Advanced Studies, Bangalore, India; 2 Department of Psychology, University of Lethbridge, Lethbridge, Canada

I thank the authors for taking into account my comments and addressing my few and minor concerns. I am happy to state that the manuscript has significantly improved. I am now glad to recommend this manuscript for indexing.

Is the study design appropriate and does the work have academic merit?

Yes

Is the work clearly and accurately presented and does it cite the current literature?

Yes

If applicable, is the statistical analysis and its interpretation appropriate?

Yes

Are all the source data underlying the results available to ensure full reproducibility?

Yes

Are the conclusions drawn adequately supported by the results?

Yes

Are sufficient details of methods and analysis provided to allow replication by others?

Yes

Reviewer Expertise:

Since 2003, my main research program focuses on the mechanisms and evolution of material culture in non-human primates (more specifically macaques), with an emphasis on stone play and stone tool use.

I confirm that I have read this submission and believe that I have an appropriate level of expertise to confirm that it is of an acceptable scientific standard.

# Peer review: Reviewer response for version 1

**DOI:** 10.21956/openreseurope.14256.r26750

**Published:** 2021-05-18

**Authors:** Kirsty Graham

**Affiliations:** 1 School of Psychology & Neuroscience, University of St Andrews, St Andrews, UK

This article is a much needed update to the question of whether nonhuman apes can make and use sharp stone tools. Previously we were left to decide whether we considered early studies of enculturated apes with incredibly small sample sizes (n=1 or 2) to be convincing evidence of this ability. Indeed, the authors take us through a step-by-step account of the training process involved in those “successful” experiments. They highlight the unusual conditions in which these apes were raised and how this may have contributed to their success. I have some specific comments about the manuscript below:

 Introduction -

 I wonder: had Kanzi been trained/taught to cut things before this experiment (e.g. using a knife)? He may have had a concept of “cutting” from past experiences.

 I agree with the authors that it’s surprising that chimpanzees haven’t been tested before, given propensity for tool-use. A valuable addition to the literature!

 Methods:

 Early in the methods the authors introduce “a sanctuary and a zoo” before naming them further down. Either name them at the initial mention or state later on that Chimfunshi Wildlife Orphanage is the sanctuary as it may not be clear to all readers.

 In the methods, it would be more helpful to give the n for the chimpanzees that were included, rather than an n for the total number of chimpanzees and then allow the reader to calculate how many the authors ended up with. Maybe change to something like “We aimed *a priori* to test all nine zoo-housed chimpanzees… However, once testing started, two zoo-housed chimpanzees were excluded from the study (n=7).” Otherwise at a cursory glance it seems that the sample size is inflated.

 Either remove “Africa” after Zambia, or add “Europe” after Norway. Could also give a more specific location for Chimfunshi in Zambia. Otherwise, it’s a very good description of the conditions for the subjects in both locations.

 Why was the “hide box” only used at the zoo and not the sanctuary?

 Flake data capture is great! Very helpful. I also appreciated the thoroughness of descriptions for the coding and analysis for interobserver reliability, as well as the power analysis for the sample size. And the ethics! A lot to commend about how clear this section is.

 Results and Discussion –

 These sections were clearly written and easy to follow. The comparison to the capuchin study is very interesting, and presented in a way that is clearly relevant for the current study.

Is the study design appropriate and does the work have academic merit?

Yes

Is the work clearly and accurately presented and does it cite the current literature?

Yes

If applicable, is the statistical analysis and its interpretation appropriate?

Yes

Are all the source data underlying the results available to ensure full reproducibility?

Yes

Are the conclusions drawn adequately supported by the results?

Yes

Are sufficient details of methods and analysis provided to allow replication by others?

Yes

Reviewer Expertise:

Primatology, animal behaviour, gestural communication.

I confirm that I have read this submission and believe that I have an appropriate level of expertise to confirm that it is of an acceptable scientific standard.

# Response

**Published:** 2021-07-28

**Authors:** Alba Motes Rodrigo

**Affiliations:** University of Tübingen, Germany

Responses to Reviewer Kirsty Graham: 1) This article is a much-needed update to the question of whether nonhuman apes can make and use sharp stone tools. Previously we were left to decide whether we considered early studies of enculturated apes with incredibly small sample sizes (n=1 or 2) to be convincing evidence of this ability. Indeed, the authors take us through a step-by-step account of the training process involved in those “successful” experiments. They highlight the unusual conditions in which these apes were raised and how this may have contributed to their success. I have some specific comments about the manuscript below:

*-->Thank you very much for your overall positive assessment of our manuscript, the succinct summary of our paper, and all the helpful comments. We address them all below and in the manuscript. We have also changed the manuscript based on feedback we received at conferences as well as on the comments of the other reviewers.*

 2) I wonder: had Kanzi been trained/taught to cut things before this experiment (e.g. using a knife)? He may have had a concept of “cutting” from past experiences.

*-->We could not find any specific information on Kanzi’s previous experience with cutting before the stone tool experiment, but Toth and colleagues do write that Kanzi was shown how to use the flake as a cutting tool: “In the first phase of the experiment, Kanzi was shown how a stone could be fractured and how the flakes produced could be used to cut the cord…” (Toth et al., 1993; p. 83). Therefore, Kanzi had the possibility to observe and learn the use of cutting tools before he was given the opportunity to make and use a flake individually. Given Kanzi's degree of enculturation, it is possible that he benefited from the demonstrations provided.*

 3) I agree with the authors that it’s surprising that chimpanzees haven’t been tested before, given propensity for tool-use. A valuable addition to the literature!

*-->Thank you very much.*

 4) Early in the methods the authors introduce “a sanctuary and a zoo” before naming them further down. Either name them at the initial mention or state later on that Chimfunshi Wildlife Orphanage is the sanctuary as it may not be clear to all readers.

*-->Thank you for this suggestion, we have now amended the manuscript as suggested.*

 5) In the methods, it would be more helpful to give the n for the chimpanzees that were included, rather than an n for the total number of chimpanzees and then allow the reader to calculate how many the authors ended up with. Maybe change to something like “We aimed a priori to test all nine zoo-housed chimpanzees… However, once testing started, two zoo-housed chimpanzees were excluded from the study (n=7).” Otherwise at a cursory glance it seems that the sample size is inflated.

*-->Thank you, we have changed the manuscript as suggested.*

 6) Either remove “Africa” after Zambia, or add “Europe” after Norway. Could also give a more specific location for Chimfunshi in Zambia. Otherwise, it’s a very good description of the conditions for the subjects in both locations.

*-->Thank you. We have removed ‘Africa’ after Zambia and included the region in which Chimfunshi is found, which comes right before the coordinates of the sanctuary.*

 7) Why was the “hide box” only used at the zoo and not the sanctuary?

*-->The hide box was designed and built after data collection was carried out at Chimfunshi. We have now included this information in the manuscript as well.*

 8) Flake data capture is great! Very helpful. I also appreciated the thoroughness of descriptions for the coding and analysis for interobserver reliability, as well as the power analysis for the sample size. And the ethics! A lot to commend about how clear this section is. These sections were clearly written and easy to follow. The comparison to the capuchin study is very interesting, and presented in a way that is clearly relevant for the current study.

*-->Thank you very much.*

# Peer review: Reviewer response for version 1

**DOI:** 10.21956/openreseurope.14256.r26751

**Published:** 2021-05-17

**Authors:** Jean-Baptiste Leca

**Affiliations:** 1 School of Natural and Engineering Sciences, National Institute of Advanced Studies, Bangalore, India; 2 Department of Psychology, University of Lethbridge, Lethbridge, Canada

This is a well-written manuscript presenting an innovative, solid, and thorough experimental study on the ability (or lack thereof) to make and use flaked stone tools by captive, naïve, and unenculturated chimpanzees. The overarching goal of this research is to gain a better understanding of the cognitive processes underlying the emergence and evolution of lithic technologies in early hominins.

 The topic was well introduced. The background literature was appropriately reviewed and critically addressed (but see below my few minor questions). The objectives were clearly laid out and ultimately reached – even though the results obtained by the authors were largely negative in the sense that the study subjects failed to express flake-making and flake-using behaviors. The methodological procedures were clearly described and extensively explained (but see below my few minor questions). The self-replication across two populations of chimpanzees was certainly a bonus.

 Overall, the layout is logical and the manuscript reads quite well. Findings from previous research were clearly summarized and put into perspective in light of this study (but see below my few minor questions). The authors did a good job at making a relatively complex study design simple, which makes the study accessible to a non-expert audience (while maintaining the interest of experts in this field!). I have no major issues with the outline/structure and conclusion of the manuscript. The (extended) figures, tables, and underlying data are informative and relevant. This is an interesting study that clearly fits an information gap, and has significant implications for the emergence of a major milestone in human evolutionary history.

 Before I can recommend this manuscript for indexing, I just have a series of minor question/concerns (some of them are actually not central to the manuscript), which I anticipate the authors will be able to handle easily.

 Page 1 (and Discussion section) I understand the phylogenetic relevance of the comparison between *Homo sapiens* and *Pan troglodytes* in specific behavioral domains to draw inferences on the evolution of specific cognitive abilities. In the context of stone tool- *making* (probably more than in the context of stone tool *use*), I am wondering to what extent some of your analyses should control for obvious inter-species differences in hand anatomy and their consequences in terms of manual dexterity, bimanual coordination, the magnitude of manual asymmetries, and variation in independent control over the digits. If it is not possible to analytically control for these variables, could you at least please elaborate on this confounder in your Discussion section?

 Page 3: Could you please clarify the following statement about your study, by possibly making it more specific?

 “Therefore, these findings suggest that without extensive human training and demonstrations, chimpanzees cannot learn how to make or use stone tools by themselves.”

 Are you suggesting that the stone-assisted nut-cracking behavior reported in several wild communities of chimpanzees across West Africa – where this activity has existed for at least 4,300 years (Mercader *et al*., 2007; Sirianni *et al*., 2015[ref-1]) – would not have been possible without any human influence?

 Or did you mean “[…] chimpanzees cannot learn how to make or use *sharp-edged* stone tools by themselves.”?

 Page 3: Could you please specify what you mean by “some types of emulation”? What are the different forms of product-oriented copying, and among them, which ones “allow for the direct transmission of behavioural forms via the observation of [a model’s…] products”?

 Page 4 (or page 9): When explaining the severe limitations of research on encultured great apes, maybe you could cite a couple of relevant studies, such as Suddendorf & Whiten (2001: Psychol Bull)[ref-2]. In their paper, they stated that “Enculturation does have the potential to modify the cognitive abilities of apes” (p. 644). They found that these modifications concern the domains of both physical cognition (such as hidden displacement of objects but also tool use) and social cognition (such mind reading, including attributing intentions to others and imitation).

 Alternatively, maybe you could elaborate on the study by Furlong *et al*. (2008) that you just cited. Even though their experiments are not about stone tool-making/use, their methodology systematically compared the performance of non-enculturated, semi-enculturated, and fully-enculturated captive chimps in three raking tool tasks, and their results provided the first empirical evidence for the differential effects of enculturation on subsequent tool use capacities in captive chimpanzees.

 Page 4: In light of your present results (about the failure to make and use flaked stone tools by naïve and unenculturated chimpanzees), how do you interpret positive findings of flake manufacture and use in untrained, unenculturated capuchin monkeys (Westergaard & Suomi, 1994) in an experimental paradigm that was similar to that used with chimpanzees? I understand your brief and speculative explanation in the Discussion section about “different genetic predispositions for stone manipulation in capuchins and chimpanzees” (page 10). However, considering the prevalence and diversity of stone-assisted percussive behaviors in wild and captive chimpanzees, I have to admit that I am not fully convinced by the interpretation that chimpanzees would be less prone to stone manipulation than capuchin monkeys. Could you please elaborate on this argument by using further empirical evidence from the literature?

 Page 5: At both study sites, you stated that “rocks” were available in the outdoor enclosure. Could you please be more specific about the exposure to these obviously relevant lithic materials? Were they loose stones or embedded substrates? If the former, would you have an estimation of the density, average size, types of stones? Were the chimpanzees ever observed manipulating them? If so, what type of manipulation are we talking about here? (e.g., exploratory, playful, instrumental/tool use?)

 Page 5: At both study sites, you stated that the chimpanzees were “familiar with test apparatuses in general” and were even provisioned with “puzzle boxes”. Could you please be more specific about the nature of these test apparatuses and puzzle boxes, other than the ones you provided to these study subjects?

 Page 8: In the Methods, you first wrote “a second coder” and then, in the next sentence, you wrote “Two naïve coders”. Could you please clarify? Was there one second coder at one study site and another second coder at the other study site?

Is the study design appropriate and does the work have academic merit?

Yes

Is the work clearly and accurately presented and does it cite the current literature?

Yes

If applicable, is the statistical analysis and its interpretation appropriate?

Yes

Are all the source data underlying the results available to ensure full reproducibility?

Yes

Are the conclusions drawn adequately supported by the results?

Yes

Are sufficient details of methods and analysis provided to allow replication by others?

Yes

Reviewer Expertise:

Since 2003, my main research program focuses on the mechanisms and evolution of material culture in non-human primates (more specifically macaques), with an emphasis on stone play and stone tool use.

I confirm that I have read this submission and believe that I have an appropriate level of expertise to confirm that it is of an acceptable scientific standard.

# Response

**Published:** 2021-07-28

**Authors:** Alba Motes Rodrigo

**Affiliations:** University of Tübingen, Germany

Responses to Reviewer Jean-Baptiste Leca: Jean-Baptiste Leca:

 1) This is a well-written manuscript presenting an innovative, solid, and thorough experimental study on the ability (or lack thereof) to make and use flaked stone tools by captive, naïve, and unenculturated chimpanzees. The overarching goal of this research is to gain a better understanding of the cognitive processes underlying the emergence and evolution of lithic technologies in early hominins. The topic was well introduced. The background literature was appropriately reviewed and critically addressed (but see below my few minor questions). The objectives were clearly laid out and ultimately reached – even though the results obtained by the authors were largely negative in the sense that the study subjects failed to express flake-making and flake-using behaviors. The methodological procedures were clearly described and extensively explained (but see below my few minor questions). The self-replication across two populations of chimpanzees was certainly a bonus. Overall, the layout is logical and the manuscript reads quite well. Findings from previous research were clearly summarized and put into perspective in light of this study (but see below my few minor questions). The authors did a good job at making a relatively complex study design simple, which makes the study accessible to a non-expert audience (while maintaining the interest of experts in this field!). I have no major issues with the outline/structure and conclusion of the manuscript. The (extended) figures, tables, and underlying data are informative and relevant. This is an interesting study that clearly fits an information gap, and has significant implications for the emergence of a major milestone in human evolutionary history.

*-->Thank you very much for the overall positive assessment of our study and for your comments, which we address below and in the manuscript. We have also changed the manuscript based on the feedback we received at conferences as well as on the comments of the other reviewers.*

 2) Before I can recommend this manuscript for indexing, I just have a series of minor question/concerns (some of them are actually not central to the manuscript), which I anticipate the authors will be able to handle easily. Page 1 (and Discussion section) I understand the phylogenetic relevance of the comparison between Homo sapiens and Pan troglodytes in specific behavioral domains to draw inferences on the evolution of specific cognitive abilities. In the context of stone tool-making (probably more than in the context of stone tool use), I am wondering to what extent some of your analyses should control for obvious inter-species differences in hand anatomy and their consequences in terms of manual dexterity, bimanual coordination, the magnitude of manual asymmetries, and variation in independent control over the digits. If it is not possible to analytically control for these variables, could you at least please elaborate on this confounder in your Discussion section?

*-->Thank you for pointing this out. We agree that* *the different hand anatomies of chimpanzees and Homo species are an important factor limiting the comparisons that can be made between these species in terms of the specific precision grips that may have been employed for stone tool making. Previous studies (Rolian et al., 2011) have found that chimpanzee hand morphology is not ideal for tasks that involved forceful oppositions of the thumb and the other fingers in precision grips, as it is the case in stone tool making. Therefore, if/when chimpanzees engaged in stone tool making, we would not expect them to use forceful precision grips. Instead, similarly to how wild chimpanzees and captive bonobos use stone tools (Toth et al. 1993 J Archaeol Sci), we would have expected the captive chimpanzees to produce sharp-edged stones using power grips. In our study, the cores provided to the chimpanzees were prepared in order to allow detachment of sharp stones from the edges even when holding a hammer with a power grip. In other words, having the ability to apply precision grips was not necessary for succeeding in the task. Consequently, we agree that the hammer grips potentially used by the chimpanzees would not have been comparable in all respects to those of Homo species. However, the question we were addressing with our experiments was whether chimpanzees would intentionally make sharp-edged stones (regardless of technique) to use them as tools, not whether they would use stone tools in the same way as Homo species did. We have added this information to the revised version of the manuscript.*

 3) Page 3: Could you please clarify the following statement about your study, by possibly making it more specific?

 “Therefore, these findings suggest that without extensive human training and/or demonstrations, chimpanzees cannot learn how to make or use stone tools by themselves.”

 Are you suggesting that the stone-assisted nut-cracking behavior reported in several wild communities of chimpanzees across West Africa – where this activity has existed for at least 4,300 years (Mercader et al., 2007; Sirianni et al., 2015 1) – would not have been possible without any human influence?

 Or did you mean “[…] chimpanzees cannot learn how to make or use sharp-edged stone tools by themselves.”?

*-->Thank you for pointing out this error. Indeed, we meant ‘sharp-edged stone tools’ and have now corrected this in the manuscript.*

 4) Page 3: Could you please specify what you mean by “some types of emulation”? What are the different forms of product-oriented copying, and among them, which ones “allow for the direct transmission of behavioural forms via the observation of [a model’s…] products”?

*-->We have now specified in the manuscript that we mean by social learning mechanisms such as “end-state emulation" (where individuals attend to the environmental changes produced by the behaviour, not the actions themselves).*

 5) Page 4 (or page 9): When explaining the severe limitations of research on encultured great apes, maybe you could cite a couple of relevant studies, such as Suddendorf & Whiten (2001: Psychol Bull) 2. In their paper, they stated that “Enculturation does have the potential to modify the cognitive abilities of apes” (p. 644). They found that these modifications concern the domains of both physical cognition (such as hidden displacement of objects but also tool use) and social cognition (such mind reading, including attributing intentions to others and imitation).

*-->Thank you for your suggestion. We have now added this reference to the manuscript when we discuss the impact of enculturation on apes.*

 6) Alternatively, maybe you could elaborate on the study by Furlong et al. (2008) that you just cited. Even though their experiments are not about stone tool-making/use, their methodology systematically compared the performance of non-enculturated, semi-enculturated, and fully-enculturated captive chimps in three raking tool tasks, and their results provided the first empirical evidence for the differential effects of enculturation on subsequent tool use capacities in captive chimpanzees. *-->Thank you. We have added more information about the study by Furlong et al. (2008) in the new version of the manuscript.*

 7) Page 4: In light of your present results (about the failure to make and use flaked stone tools by naïve and unenculturated chimpanzees), how do you interpret positive findings of flake manufacture and use in untrained, unenculturated capuchin monkeys (Westergaard & Suomi, 1994) in an experimental paradigm that was similar to that used with chimpanzees? I understand your brief and speculative explanation in the Discussion section about “different genetic predispositions for stone manipulation in capuchins and chimpanzees” (page 10). However, considering the prevalence and diversity of stone-assisted percussive behaviors in wild and captive chimpanzees, I have to admit that I am not fully convinced by the interpretation that chimpanzees would be less prone to stone manipulation than capuchin monkeys. Could you please elaborate on this argument by using further empirical evidence from the literature?

*-->Wild capuchins have been observed using stones in a variety of foraging behaviors (digging, nut-cracking, and cracking molluscs and crustaceans in coastal environments; Falótico et al. 2017 Sci Rep, Barret et al. 2018 RSOS; Visalberghi et al. 2005 Science) as well as during sexual displays (Falótico and Ottoni, 2013 PLOS ONE). Captive capuchins show similar tool using abilities to their wild counterparts and develop stone tool-use behaviors in the absence of social demonstrations (Westergaard and Suomi, 1993 J Hum Evol; Ottoni and Mannu 2001 Int J Primatol; Visalberghi 1987 Folia Primatol). Furthermore, recent findings in other species of stone tool using primates (e.g., long-tailedmacaques) suggest that individuals with specific phenotypes are more likely to engage in stone tool using behaviors (Gumert et al. 2019 Behavior). On the contrary, although several populations of chimpanzees have been observed using stone tools, this behavior is by no means widespread across chimpanzee populations. Furthermore, captive populations of chimpanzees seem to only develop stone tool using behaviors after dedicated training and/or demonstrations (Sumita et al. 1985 Primates; Neadle et al. 2019 PeerJ; although there are also some unclear cases which may show individual learning instead; e.g. Marshall-Pescini et al., 2005 J Comp Psychol). Given the distribution of stone tool use among chimpanzee and capuchin populations, we (and others, Hayachi 2015 Phil Trans B) believe that, similar to other tool using taxa such as otters (Fujii et al., 2017 Behav Ecology) and crows (Kenward et al. 2006 Anim Behav), capuchins (rather than chimpanzee) are likely to have some genetic predisposition(s) for stone manipulation.*

 8) Page 5: At both study sites, you stated that “rocks” were available in the outdoor enclosure. Could you please be more specific about the exposure to these obviously relevant lithic materials? Were they loose stones or embedded substrates? If the former, would you have an estimation of the density, average size, types of stones? Were the chimpanzees ever observed manipulating them? If so, what type of manipulation are we talking about here? (e.g., exploratory, playful, instrumental/tool use?)

*-->Thank you for pointing this out. At Kristiansand zoo all stones had been removed from the outdoor enclosure of the chimpanzees by the keepers to prevent the chimpanzees from throwing them at the visitors. Therefore, stones were only available at Chimfunshi. The outdoor area at Chimfunshi measures approx. 75 square meters and it contained a few small pebble-like stones. Although we did not collect systematic data on the size or abundance of stones, we never observed the chimpanzees’ manipulating these small pebbles during testing. However, we agree that it would have been a good idea to systematically observe if and how the chimpanzees manipulated the stones available in their environment.*

 9) Page 5: At both study sites, you stated that the chimpanzees were “familiar with test apparatuses in general” and were even provisioned with “puzzle boxes”. Could you please be more specific about the nature of these test apparatuses and puzzle boxes, other than the ones you provided to these study subjects?

*-->At Chimfunshi the chimpanzees were regularly provided with baited cardboard tubes that were folded at the edges. The chimpanzees then had to use small twigs or their hands and teeth to access the tubes and retrieve the food, as a form of enrichment. At Kristiansand zoo the chimpanzees had several enrichment devices in the indoor enclosure aimed at promoting the use of tools such as a perforated hanging log baited with honey and berries and an automatic puzzle feeder that periodically released nuts into a maze that the chimpanzees could obtain using tools (Motes-Rodrigo et al. 2019 Primates). We have now added this information to the manuscript.*

 10) Page 8: In the Methods, you first wrote “a second coder” and then, in the next sentence, you wrote “Two naïve coders”. Could you please clarify? Was there one second coder at one study site and another second coder at the other study site?

*-->Thank you. We had two, different, second coders in total, one for each testing institution. We have now clarified this in the manuscript.*

# Peer review: Reviewer response for version 1

**DOI:** 10.21956/openreseurope.14256.r26632

**Published:** 2021-04-15

**Authors:** Mark Moore

**Affiliations:** 1 Archaeology and Palaeoanthropology, University of New England (UNE), Armidale, Australia

This is an important study into the ability of chimpanzees to spontaneously make and use stone flakes, and I recommend indexing with minor revisions.

 The topic is important because some researchers see the emergence of stone-flaking as cognitively insignificant, while others see it as a sort of ‘cognitive Rubicon’. An independent assessment of these competing models is possible through what the authors refer to as ‘cognitive cladistics’—by studying the capabilities of the great apes, our closest living primate relatives. The authors assess prior experiments in great ape stone-flaking, and conclude that the results are of limited use for two reasons: 1) the tested apes were enculturated into human ways of learning because they were reared in a human cultural environment; and 2) stone-flaking and use was demonstrated to the tested apes. The behaviours observed in prior studies were therefore not ‘spontaneous’, and did not track ‘naturally developing abilities’, and are therefore of limited use in assessing the emergence of stone flaking. The authors’ experiments in this paper were with unenculturated chimpanzees, and without prior demonstration, in order to test the hypothesis that flake manufacture and use were within the ‘natural individual cognitive reach of apes’.

 A premise of the present study is that the learning environment was key to the copying of stone flaking behaviour, as summarised in the paper’s introduction. Therefore, the authors argue, the prior experiments were of limited value because the subjects were deliberately and extensively enculturated by their handlers. However, Eren *et al.* (2019, ^1^ not cited by the authors) suggest that it is ‘incongruous’ that the apes—particularly Kanzi, a bonobo—in these prior experiments were not able to mimic Oldowan stone tools given hominoid’s technological propensities, suggesting instead that the apes were actually working in an impoverished learning environment. Eren *et al*. suggest that apes may indeed have the necessary cognition to make Oldowan stone tools, but that the ‘misdirection’ inherent in the demonstrations by human experimenters compromised the ape’s learning; and indeed, Eren *et al*. suggest that the demonstrations were ‘minimal’ (Eren *et al*. 2019:313), in contradiction to the present authors’ interpretation of the same studies (although I note that the present authors express more ambivalence about the importance of ‘demonstrations’ in the discussion section than in the introduction). The authors should consider and respond to Eren *et al*.’s conclusion in developing the background to their experiments.

 The experimental design followed aspects of prior work on this topic by using two types of baited boxes, and provisioning the rooms with one or more indentors (hammerstones) and a high-quality knapping stone with appropriate angles for flaking. In one set of experiments, the hammerstone was made of concrete and chained to the metal bars of the cage, and the core was secured in wire mesh. This effectively prevented a full and free manipulation of these objects, and would have stopped the sort of stone-fracturing events documented in Kanzi; that is, the chimpanzees in this set of the authors’ experiments were not given full range to discover the affordances offered by the stones. However, the chimpanzees in the other set of experiments were given the stones unimpeded by chains and wire, yet those chimpanzees also did not make Kanzi-like stone-flaking manipulations. Because of this, although perhaps not ideal, I am happy with accepting this aspect of the experimental design. I note that all of the chimpanzees were given free manipulation of the provided flake in certain experimental iterations, yet did not discover the affordance offered by the sharp edge. The experimental design, controls, and data collection effectively support the authors’ experimental conclusions that chimpanzees do not spontaneously flake stone to solve ecological problems.

 One aspect of the discussion needs to be further elaborated, however. The authors state that the chimpanzees ‘demonstrated an understanding of the requirements of the task’ to remove the treats from the two types of containers, and were ‘motivated to open the puzzle boxes’. Their evidence for this is the observation that the chimpanzees ‘frequently interacted with’ the lock/hide mechanisms. However, the authors’ observations are vague and, as given, are insufficient for inferring ‘understanding’ and ‘motivation’. More detail is necessary to support this point, as ‘understanding’ and ‘motivation’ are key in the epistemological chain that links the possible recognition of tool affordances to a goal. The ways that the chimpanzees used other objects in attempts to gain access to the boxes—referred to only briefly—may provide the necessary information if developed in more detail. How, specifically, were those objects used, and what does this say about the characteristics the chimpanzees thought those objects had, but were (apparently) not perceived in the stones? Perhaps there’s a degree of object familiarity at play here.

 Under ‘coding’, the authors should clarify that the tool-use referred to involved other objects the chimpanzees brought into the room, and not stone tools.

Is the study design appropriate and does the work have academic merit?

Yes

Is the work clearly and accurately presented and does it cite the current literature?

Partly

If applicable, is the statistical analysis and its interpretation appropriate?

Yes

Are all the source data underlying the results available to ensure full reproducibility?

Yes

Are the conclusions drawn adequately supported by the results?

Yes

Are sufficient details of methods and analysis provided to allow replication by others?

Yes

Reviewer Expertise:

Stone tools, hominin cognitive evolution, the design space of stone flaking.

I confirm that I have read this submission and believe that I have an appropriate level of expertise to confirm that it is of an acceptable scientific standard.

## References

[1] : Underestimating Kanzi? Exploring Kanzi-Oldowan comparisons in light of recent human stone tool replication. *Evol Anthropol* .2020;29(6) : 10.1002/evan.21858 310-316 10.1002/evan.21858 32857904

# Response

**Published:** 2021-07-28

**Authors:** Alba Motes Rodrigo

**Affiliations:** University of Tübingen, Germany

Responses to Reviewer Mark Moore:

 1) This is an important study into the ability of chimpanzees to spontaneously make and use stone flakes, and I recommend indexing with minor revisions. The topic is important because some researchers see the emergence of stone-flaking as cognitively insignificant, while others see it as a sort of ‘cognitive Rubicon’. An independent assessment of these competing models is possible through what the authors refer to as ‘cognitive cladistics’—by studying the capabilities of the great apes, our closest living primate relatives. The authors assess prior experiments in great ape stone-flaking, and conclude that the results are of limited use for two reasons: 1) the tested apes were enculturated into human ways of learning because they were reared in a human cultural environment; and 2) stone-flaking and use was demonstrated to the tested apes. The behaviours observed in prior studies were therefore not ‘spontaneous’, and did not track ‘naturally developing abilities’, and are therefore of limited use in assessing the emergence of stone flaking. The authors’ experiments in this paper were with unenculturated chimpanzees, and without prior demonstration, in order to test the hypothesis that flake manufacture and use were within the ‘natural individual cognitive reach of apes’.

*-->Thank you very much for the overall positive assessment of the study. We have responded to your comments below and in the manuscript. We have also changed the manuscript based on feedback we received at conferences as well as on the comments of the other reviewers.* *We agree with the summary and we would only like to clarify that the enculturation process can not only enhance/install certain social learning capabilities in apes, but can also increase their innovative capabilities. Enculturation can enable apes to learn behavioural know-how from demonstrations whilst at the same time influence their individual learning abilities. In such cases, enculturated apes could be able to innovate behaviors (such as sharp-edged stone tool making and use) beyond what unenculturated individuals could have learned on their own. For example, the first successful sharp-edged stone tool production technique used by Kanzi (throwing) had not been demonstrated to him. We have now clarified this point in our manuscript.*

 2) A premise of the present study is that the learning environment was key to the copying of stone flaking behaviour, as summarised in the paper’s introduction. Therefore, the authors argue, the prior experiments were of limited value because the subjects were deliberately and extensively enculturated by their handlers. However, Eren et al. (2019, 1 not cited by the authors) suggest that it is ‘incongruous’ that the apes—particularly Kanzi, a bonobo—in these prior experiments were not able to mimic Oldowan stone tools given hominoid’s technological propensities, suggesting instead that the apes were actually working in an impoverished learning environment. Eren et al. suggest that apes may indeed have the necessary cognition to make Oldowan stone tools, but that the ‘misdirection’ inherent in the demonstrations by human experimenters compromised the ape’s learning; and indeed, Eren et al. suggest that the demonstrations were ‘minimal’ (Eren et al. 2019:313), in contradiction to the present authors’ interpretation of the same studies (although I note that the present authors express more ambivalence about the importance of ‘demonstrations’ in the discussion section than in the introduction). The authors should consider and respond to Eren et al.’s conclusion in developing the background to their experiments.

*-->Thank you for bringing Eren et al.s’ paper to our attention, which we have now cited in the manuscript. Although we believe that the discussion of Eren et al. is interesting to better understand the stone tool making and using abilities of enculturated apes, we believe that the relevance of their conclusion for our study is limited. Eren et al. discuss the fact that whilst Kanzi made stone tools, he did not use the techniques used by early hominins nor did he produce the same type of flakes found in the Oldowan archaeological record. Eren et al. argue that this is because Kanzi did not have access to demonstrators with sufficient knowledge of how to make Oldowan flakes. Although Eren et al.'s arguments are a valuable contribution to the discussion of Kanzi's proficiency – and the general proficiency of enculturated apes –  in sharp-stone tool making, the focus of our study was on whether unenculturated chimpanzees would spontaneously make and use sharp-edged stones in the absence of demonstrations. Therefore, the conclusions of Eren et al. are of very limited applicability for our findings. Furthermore, we believe that the fact that Kanzi first used a sharp-edged stone tool making technique that had not been demonstrated to him contradicts the conclusion of Eren et al. that the quality of the demonstrations hindered the bonobo's performance.*

 3) The experimental design followed aspects of prior work on this topic by using two types of baited boxes, and provisioning the rooms with one or more indentors (hammerstones) and a high-quality knapping stone with appropriate angles for flaking. In one set of experiments, the hammerstone was made of concrete and chained to the metal bars of the cage, and the core was secured in wire mesh. This effectively prevented a full and free manipulation of these objects, and would have stopped the sort of stone-fracturing events documented in Kanzi; that is, the chimpanzees in this set of the authors’ experiments were not given full range to discover the affordances offered by the stones. However, the chimpanzees in the other set of experiments were given the stones unimpeded by chains and wire, yet those chimpanzees also did not make Kanzi-like stone-flaking manipulations. Because of this, although perhaps not ideal, I am happy with accepting this aspect of the experimental design. I note that all of the chimpanzees were given free manipulation of the provided flake in certain experimental iterations, yet did not discover the affordance offered by the sharp edge. The experimental design, controls, and data collection effectively support the authors’ experimental conclusions that chimpanzees do not spontaneously flake stone to solve ecological problems.

*-->We agree that it would have been preferable to provide loose hammers and cores at both institutions to allow for a broader range of manipulations and affordances, but unfortunately, we could not do so due to the institutions’ safety concerns. However, we do not fully agree with the reviewer that the set up at Kristiansand prevented the chimpanzees from performing all stone-fracturing events reported in the studies with Kanzi. According to Toth et al. (1993, J Arhaeol Sci), "Kanzi sometimes held one rock in his right hand and struck it repeatedly against another stone on the ground" (p.84) and "Kanzi had discovered on his own that he can throw a well-aimed cobble against another on the ground to produce flakes and fragments [...]" (p.85). Both of these techniques were not demonstrated to Kanzi and were equally available to the chimpanzees tested at Kristiansand zoo. The only techniques that the chimpanzees could not perform were those involving hand-held/body-stabilised cores such as in freehand knapping. Therefore, the experimental set up did not prevent the untrained, unenculturated chimpanzees at Kristiansand zoo from engaging in all stone-fracturing techniques previously innovated by Kanzi.*

 4) One aspect of the discussion needs to be further elaborated, however. The authors state that the chimpanzees ‘demonstrated an understanding of the requirements of the task’ to remove the treats from the two types of containers, and were ‘motivated to open the puzzle boxes’. Their evidence for this is the observation that the chimpanzees ‘frequently interacted with’ the lock/hide mechanisms. However, the authors’ observations are vague and, as given, are insufficient for inferring ‘understanding’ and ‘motivation’. More detail is necessary to support this point, as ‘understanding’ and ‘motivation’ are key in the epistemological chain that links the possible recognition of tool affordances to a goal. The ways that the chimpanzees used other objects in attempts to gain access to the boxes—referred to only briefly—may provide the necessary information if developed in more detail. How, specifically, were those objects used, and what does this say about the characteristics the chimpanzees thought those objects had, but were (apparently) not perceived in the stones? Perhaps there’s a degree of object familiarity at play here.

*-->Following the Reviewer’s comment, we have rephrased our results and discussion in the manuscript stating that the chimpanzees were motivated to access the food rewards as shown by their manipulations of the puzzle boxes. In addition, we have also rephrased our conclusions regarding the causal or physical understanding that the chimpanzees might have had of the puzzle boxes, as this question could not be directly answered with the data we collected. We have also added to our discussion that the fact that the chimpanzees used familiar materials as tools but not the unfamiliar stone flakes might represent a case of conservatism in the use of novel tool materials. Regarding the comment of the reviewer about the chimpanzees "understanding" the requirements of the task, we believe that the results from previous studies on the topic of chimpanzee problem solving make it possible that the chimpanzees understood the puzzle boxes provided in our study. The hide box was a transparent device where access to a food reward was prevented by a translucid silicone membrane. Taking also into consideration that chimpanzees can spontaneously solve multi-step sequential puzzles even when requiring the use of tools (e.g., Vale et al. 2021 Evolution and Human Behavior), it is unlikely that all the chimpanzees across both testing locations did not understand the requirements of the hide box. Therefore, we believe that most apes likely did “understand” the hide box. On the other hand, it is a possibility that at least some chimpanzees did not understand the requirements of the tendon box (given that this box was opaque). The reason behind using the tendon box in our experiments was that a box with a very similar design had been employed in previous flaking studies with enculturated apes (Wright 1972 Mankind; Toth et al. 1993 J Archaeol Sci), who successfully opened them using sharp-edged stone tools after watching demonstrations. Given that we wanted to provide the chimpanzees with a task as similar as possible to the previous ape flaking studies, we used boxes with similar designs as those used by Toth et al. and Wright.*

 5) Under ‘coding’, the authors should clarify that the tool-use referred to involved other objects the chimpanzees brought into the room, and not stone tools.

*--> Thank you, we have now added this information to the manuscript.*
